# Supplementary material for: Global burden and future trends of inguinal, femoral, and abdominal hernia in older adults: A systematic analysis from the Global Burden of Disease Study 2021
Source: PLoS One. 2025 Jun 6;20(6):e0323790. doi: 10.1371/journal.pone.0323790 (PMC12143580; doi:10.1371/journal.pone.0323790)
Supplement: S1 File — S2 Table. Predictions of inguinal, femoral, and abdominal hernias among older adults: age-standardized incidence, prevalence and DALYs rates and total cases to 2035 with 95% uncertainty intervals. Please refer to the additional submitted attachment for details. (DOCX) [file pone.0323790.s001.docx]

**S1 Table. Incidence, Prevalence and DALYs of Inguinal, Femoral, and Abdominal Hernias among** **Older Adults in 1990 and 2021, and AAPC from 1990 to 2021, by Countries.**

| **Location** | **Incidence** | | | | | **Prevalence** | | | | | **DALYs** | | | | |
| --- | --- | --- | --- | --- | --- | --- | --- | --- | --- | --- | --- | --- | --- | --- | --- |
|  | **Incident cases(1990)** | **ASR of Incidence(1990)** | **Incident cases(2021)** | **ASR of Incidence(2021)** | **AAPC**  **1990–2021** | **Prevalent cases(1990)** | **ASR of Prevalence(1990)** | **Prevalent cases(2021)** | **ASR of Prevalence(2021)** | **AAPC**  **1990–2021** | **DALYs cases (1990)** | **ASR of DALYs(1990)** | **DALYs cases(2021)** | **ASR of**  **DALYs( 2021)** | **AAPC**  **1990–2021** |
| **Afghanistan** | 1336(778 to 2099) | 146.87(85.48 to 231.19) | 639(385 to 988) | 77.90(47.02 to 120.94) | -2.08(-2.39 to -1.76) | 3276(2095 to 4870) | 370.20(236.64 to 549.83) | 2730(1865 to 3816) | 330.87(224.48 to 465.01) | -0.41(-0.50 to -0.32) | 377(214 to 680) | 45.61(25.57 to 85.17) | 307(175 to 510) | 37.67(21.27 to 63.26) | -0.65(-0.72 to -0.57) |
| **Albania** | 942(567 to 1452) | 375.89(226.03 to 578.86) | 1220(757 to 1826) | 201.19(124.86 to 300.94) | -2.01(-2.19 to -1.83) | 1704(1226 to 2303) | 695.83(501.56 to 939.36) | 2315(1529 to 3345) | 387.50(256.58 to 558.73) | -1.91(-2.02 to -1.79) | 260(180 to 360) | 116.53(80.60 to 161.55) | 268(165 to 418) | 47.17(29.06 to 73.26) | -2.94(-3.23 to -2.66) |
| **Algeria** | 2417(1396 to 3869) | 150.57(87.12 to 240.27) | 5003(2974 to 7909) | 111.13(66.21 to 175.10) | -0.99(-1.11 to -0.88) | 4567(2775 to 7028) | 289.17(176.95 to 442.77) | 12170(7448 to 18268) | 274.73(169.53 to 410.24) | -0.20(-0.30 to -0.11) | 414(222 to 752) | 29.36(15.39 to 58.61) | 1024(549 to 1819) | 24.56(13.14 to 44.84) | -0.59(-0.67 to -0.52) |
| **American Samoa** | 3(2 to 5) | 118.04(74.45 to 177.20) | 7(5 to 10) | 119.28(79.86 to 169.77) | 0.07(-0.01 to 0.16) | 11(8 to 14) | 435.35(316.73 to 574.43) | 27(20 to 35) | 477.09(361.56 to 614.75) | 0.33(0.25 to 0.40) | 2(1 to 3) | 77.66(49.95 to 123.23) | 5(3 to 9) | 105.13(55.84 to 165.93) | 0.99(0.37 to 1.62) |
| **Andorra** | 31(19 to 49) | 412.48(252.73 to 638.51) | 37(24 to 55) | 194.65(123.99 to 286.55) | -2.38(-2.55 to -2.21) | 28(19 to 40) | 379.87(253.90 to 544.21) | 54(37 to 77) | 275.63(187.97 to 394.21) | -1.06(-1.18 to -0.95) | 6(4 to 9) | 94.02(57.56 to 145.43) | 11(7 to 18) | 54.42(32.82 to 85.13) | -1.83(-2.23 to -1.43) |
| **Angola** | 394(234 to 600) | 94.14(55.89 to 143.35) | 1218(773 to 1798) | 95.62(60.41 to 140.94) | -0.02(-0.25 to 0.22) | 2435(1745 to 3267) | 604.10(434.11 to 806.89) | 6451(4825 to 8307) | 523.67(391.55 to 674.77) | -0.53(-0.61 to -0.45) | 547(273 to 964) | 155.49(76.44 to 282.41) | 1447(795 to 2456) | 136.76(73.49 to 239.75) | -0.40(-0.48 to -0.32) |
| **Antigua and Barbuda** | 8(5 to 12) | 117.87(74.94 to 176.80) | 14(9 to 20) | 99.68(64.33 to 143.87) | -0.56(-0.74 to -0.37) | 28(20 to 37) | 411.97(295.27 to 551.67) | 42(30 to 56) | 306.88(222.26 to 403.59) | -0.99(-1.08 to -0.89) | 6(5 to 7) | 84.75(67.25 to 105.11) | 9(7 to 11) | 68.08(52.74 to 86.62) | -0.77(-1.66 to 0.14) |
| **Argentina** | 5075(3140 to 7822) | 119.15(73.76 to 183.58) | 7862(5216 to 11116) | 110.29(73.14 to 156.19) | -0.22(-0.43 to -0.01) | 15374(11080 to 20383) | 364.40(263.38 to 482.21) | 24378(18396 to 31537) | 340.37(255.98 to 441.44) | -0.22(-0.25 to -0.18) | 3746(3022 to 4567) | 94.10(76.06 to 114.44) | 5628(4570 to 6834) | 77.29(62.65 to 94.04) | -0.60(-0.97 to -0.23) |
| **Armenia** | 595(392 to 856) | 166.56(108.36 to 242.00) | 1067(717 to 1504) | 174.40(116.90 to 246.45) | 0.24(0.04 to 0.43) | 1904(1383 to 2528) | 586.46(429.81 to 769.77) | 3085(2304 to 4004) | 520.83(390.65 to 673.14) | -0.38(-0.41 to -0.34) | 409(330 to 509) | 130.33(105.30 to 161.42) | 591(467 to 724) | 101.88(80.57 to 124.58) | -0.91(-1.96 to 0.16) |
| **Australia** | 4204(2749 to 6097) | 161.04(105.30 to 233.68) | 7292(4780 to 10582) | 122.89(79.80 to 179.75) | -0.86(-0.91 to -0.80) | 9147(6458 to 12459) | 353.13(249.25 to 480.93) | 13466(9534 to 18633) | 223.09(155.92 to 311.99) | -1.47(-1.51 to -1.44) | 2017(1630 to 2475) | 80.96(65.44 to 99.07) | 3787(2956 to 4651) | 58.72(45.73 to 72.66) | -0.96(-1.74 to -0.16) |
| **Austria** | 5428(3132 to 8250) | 350.77(202.09 to 535.23) | 4832(3097 to 7181) | 224.50(142.67 to 335.81) | -1.42(-1.55 to -1.30) | 8233(6123 to 10737) | 527.64(389.68 to 691.83) | 7543(5182 to 10717) | 346.51(235.25 to 497.57) | -1.38(-1.41 to -1.34) | 1639(1333 to 2005) | 102.73(82.83 to 126.36) | 1387(1038 to 1796) | 55.64(41.07 to 73.60) | -1.87(-2.19 to -1.55) |
| **Azerbaijan** | 696(425 to 1038) | 110.36(66.27 to 166.66) | 1181(752 to 1740) | 81.55(51.52 to 120.51) | -0.97(-1.17 to -0.77) | 1968(1348 to 2709) | 324.57(222.65 to 446.70) | 3709(2484 to 5145) | 266.18(179.91 to 366.69) | -0.66(-0.73 to -0.60) | 299(200 to 457) | 51.82(34.70 to 79.72) | 467(299 to 701) | 37.13(23.62 to 56.06) | -1.06(-1.24 to -0.88) |
| **Bahamas** | 26(16 to 39) | 146.75(92.06 to 219.33) | 49(32 to 70) | 95.51(63.50 to 136.45) | -1.36(-1.42 to -1.30) | 92(68 to 119) | 511.96(379.50 to 661.82) | 170(127 to 221) | 335.66(252.13 to 434.39) | -1.36(-1.42 to -1.31) | 21(17 to 26) | 121.50(97.73 to 147.66) | 40(31 to 51) | 85.19(66.56 to 107.68) | -1.23(-2.05 to -0.41) |
| **Bahrain** | 40(24 to 62) | 209.11(127.11 to 321.86) | 156(95 to 240) | 138.33(86.27 to 209.86) | -1.29(-1.41 to -1.17) | 74(49 to 106) | 414.89(284.48 to 584.38) | 298(182 to 459) | 282.39(179.82 to 421.50) | -1.27(-1.38 to -1.16) | 10(7 to 13) | 62.80(44.28 to 86.84) | 40(26 to 58) | 53.10(35.13 to 76.63) | -0.54(-1.01 to -0.06) |
| **Bangladesh** | 3793(2158 to 5988) | 69.12(39.37 to 108.86) | 13363(7818 to 21156) | 76.21(44.70 to 120.39) | 0.32(0.09 to 0.56) | 17612(11675 to 24962) | 323.08(215.41 to 455.97) | 52768(32781 to 77392) | 302.15(188.78 to 441.60) | -0.28(-0.37 to -0.18) | 15971(9474 to 25922) | 313.56(185.19 to 508.10) | 16359(9375 to 29811) | 105.68(60.66 to 192.07) | -3.44(-3.75 to -3.14) |
| **Barbados** | 51(32 to 74) | 137.77(88.14 to 202.55) | 74(50 to 107) | 105.57(70.88 to 152.82) | -0.86(-1.02 to -0.71) | 168(124 to 217) | 448.31(328.37 to 584.64) | 232(173 to 304) | 332.20(247.18 to 434.58) | -0.99(-1.04 to -0.94) | 42(34 to 52) | 108.10(87.50 to 132.66) | 49(36 to 65) | 71.45(52.81 to 93.80) | -1.28(-1.68 to -0.88) |
| **Belarus** | 5325(3250 to 8048) | 311.08(190.57 to 469.27) | 4334(2821 to 6382) | 194.42(126.82 to 285.70) | -1.51(-1.60 to -1.42) | 8098(5640 to 11099) | 485.29(341.37 to 659.26) | 8564(6088 to 11809) | 388.69(278.07 to 532.73) | -0.71(-0.76 to -0.66) | 1459(1147 to 1845) | 89.40(70.47 to 112.59) | 1439(1046 to 1903) | 66.69(48.50 to 87.95) | -0.97(-1.29 to -0.65) |
| **Belgium** | 7698(4364 to 12028) | 377.63(213.75 to 590.87) | 5331(3365 to 7994) | 187.03(117.02 to 282.54) | -2.24(-2.42 to -2.07) | 9899(7101 to 13310) | 484.13(345.76 to 653.20) | 8306(5569 to 11757) | 285.52(188.58 to 408.93) | -1.68(-1.83 to -1.54) | 1792(1436 to 2228) | 88.08(70.41 to 109.60) | 1810(1365 to 2314) | 53.91(40.37 to 70.15) | -1.49(-2.00 to -0.98) |
| **Belize** | 14(8 to 21) | 119.24(72.94 to 181.02) | 33(22 to 48) | 94.39(62.27 to 135.46) | -0.76(-0.83 to -0.70) | 51(36 to 67) | 443.00(319.12 to 587.70) | 120(90 to 156) | 343.14(258.58 to 440.72) | -0.85(-0.99 to -0.72) | 12(9 to 16) | 104.79(77.56 to 141.50) | 33(26 to 41) | 98.78(79.13 to 121.94) | -0.05(-1.34 to 1.26) |
| **Benin** | 237(145 to 362) | 98.09(60.04 to 149.65) | 516(331 to 761) | 87.56(56.02 to 128.88) | -0.47(-0.80 to -0.15) | 1220(874 to 1628) | 514.16(368.72 to 685.27) | 2480(1870 to 3205) | 429.99(324.96 to 554.52) | -0.64(-0.73 to -0.55) | 365(201 to 914) | 163.86(89.19 to 415.26) | 715(379 to 1763) | 137.43(72.10 to 345.28) | -0.57(-0.63 to -0.51) |
| **Bermuda** | 14(9 to 20) | 174.88(115.83 to 250.18) | 23(15 to 34) | 130.92(86.49 to 193.72) | -0.92(-0.96 to -0.88) | 43(32 to 57) | 545.49(405.96 to 717.82) | 52(37 to 70) | 289.77(206.97 to 393.35) | -2.03(-2.07 to -1.99) | 11(9 to 13) | 147.09(117.60 to 178.49) | 14(11 to 18) | 73.15(56.70 to 93.50) | -2.27(-2.93 to -1.61) |
| **Bhutan** | 32(19 to 48) | 115.60(69.77 to 174.52) | 77(49 to 112) | 105.31(66.91 to 154.35) | -0.29(-0.32 to -0.26) | 189(135 to 253) | 714.25(513.80 to 952.98) | 290(207 to 389) | 402.42(287.37 to 539.34) | -1.85(-1.95 to -1.74) | 58(31 to 134) | 247.20(131.78 to 554.98) | 86(48 to 200) | 125.00(69.81 to 290.24) | -2.18(-2.30 to -2.07) |
| **Bolivia (Plurinational State of)** | 603(357 to 935) | 154.94(91.31 to 240.32) | 1313(845 to 1944) | 114.57(73.62 to 169.31) | -0.95(-1.03 to -0.87) | 3135(2277 to 4158) | 817.14(593.76 to 1083.37) | 5674(4249 to 7326) | 500.95(375.23 to 646.70) | -1.59(-1.66 to -1.52) | 752(393 to 1183) | 211.04(108.96 to 334.03) | 1257(740 to 1955) | 120.09(69.87 to 187.85) | -1.83(-1.96 to -1.69) |
| **Bosnia and Herzegovina** | 1724(1170 to 2484) | 344.62(232.18 to 500.54) | 1831(1190 to 2632) | 214.60(139.42 to 308.58) | -1.55(-1.82 to -1.28) | 5280(4176 to 6567) | 1112.73(883.78 to 1378.94) | 3506(2476 to 4752) | 414.16(292.66 to 560.97) | -3.13(-3.20 to -3.06) | 862(661 to 1096) | 193.25(148.33 to 245.27) | 539(378 to 747) | 64.31(45.11 to 89.02) | -3.48(-3.67 to -3.29) |
| **Botswana** | 49(29 to 74) | 71.38(42.61 to 107.43) | 121(79 to 178) | 70.41(45.65 to 103.21) | -0.01(-0.05 to 0.03) | 311(227 to 413) | 459.65(336.10 to 607.75) | 589(448 to 754) | 347.43(264.31 to 444.15) | -0.91(-1.00 to -0.83) | 95(58 to 165) | 163.98(98.06 to 290.85) | 151(96 to 229) | 101.58(64.46 to 155.21) | -1.61(-2.19 to -1.03) |
| **Brazil** | 21981(13813 to 32280) | 200.72(125.95 to 295.01) | 44198(28871 to 63713) | 138.41(90.41 to 199.39) | -1.20(-1.25 to -1.14) | 98290(76156 to 124054) | 912.44(707.31 to 1150.24) | 172931(134757 to 216538) | 545.38(425.62 to 681.95) | -1.64(-1.70 to -1.58) | 16730(14209 to 19574) | 166.69(142.09 to 193.72) | 49619(43185 to 56195) | 160.64(139.72 to 181.70) | -0.11(-0.34 to 0.14) |
| **Brunei Darussalam** | 17(10 to 27) | 153.18(88.49 to 246.41) | 54(32 to 84) | 127.20(75.45 to 196.87) | -0.97(-2.90 to 0.99) | 23(14 to 35) | 209.14(126.46 to 320.42) | 72(44 to 109) | 177.74(112.77 to 265.07) | -0.53(-0.69 to -0.38) | 4(3 to 6) | 41.45(25.08 to 62.99) | 10(6 to 15) | 30.93(19.72 to 45.29) | -0.98(-1.17 to -0.78) |
| **Bulgaria** | 9382(5930 to 13760) | 528.03(332.50 to 775.79) | 4555(2904 to 6803) | 234.17(149.31 to 350.00) | -2.59(-2.69 to -2.48) | 21774(17113 to 27158) | 1272.84(1001.22 to 1585.87) | 9494(6639 to 13049) | 486.05(340.61 to 668.44) | -3.10(-3.28 to -2.92) | 2879(2264 to 3570) | 191.78(153.06 to 235.24) | 1270(952 to 1686) | 65.31(48.92 to 86.70) | -3.38(-3.84 to -2.92) |
| **Burkina Faso** | 599(372 to 892) | 110.82(68.60 to 165.49) | 1275(805 to 1883) | 116.60(73.42 to 172.02) | 0.08(-0.08 to 0.23) | 3182(2322 to 4203) | 613.99(449.66 to 808.45) | 6499(4778 to 8570) | 612.43(452.46 to 804.51) | -0.07(-0.18 to 0.04) | 713(407 to 1669) | 156.12(87.30 to 373.57) | 1543(846 to 3910) | 160.31(86.81 to 409.29) | 0.10(-0.07 to 0.26) |
| **Burundi** | 272(161 to 420) | 98.86(58.68 to 152.68) | 624(385 to 945) | 109.58(67.46 to 165.57) | 0.22(-0.07 to 0.52) | 1270(888 to 1740) | 459.64(321.24 to 630.54) | 3357(2438 to 4475) | 593.12(430.97 to 789.99) | 0.84(0.78 to 0.91) | 435(231 to 919) | 174.98(91.55 to 386.44) | 805(440 to 1843) | 170.53(91.31 to 399.81) | -0.07(-0.15 to 0.01) |
| **Cabo Verde** | 24(15 to 36) | 81.76(49.94 to 123.89) | 51(33 to 76) | 95.07(61.99 to 141.29) | 0.42(0.20 to 0.65) | 114(81 to 153) | 387.11(273.71 to 520.20) | 181(131 to 240) | 338.93(247.20 to 449.01) | -0.59(-0.73 to -0.45) | 27(13 to 82) | 89.08(42.67 to 272.18) | 43(25 to 88) | 84.40(48.80 to 172.74) | -0.15(-0.40 to 0.11) |
| **Cambodia** | 623(378 to 961) | 118.72(71.97 to 182.97) | 1559(1023 to 2241) | 103.45(67.73 to 148.41) | -0.46(-0.59 to -0.32) | 1924(1367 to 2599) | 384.26(273.37 to 518.48) | 4759(3539 to 6151) | 327.47(244.82 to 421.92) | -0.54(-0.60 to -0.48) | 717(413 to 1303) | 157.59(90.35 to 288.22) | 1542(925 to 2446) | 120.30(71.68 to 190.12) | -0.86(-0.91 to -0.81) |
| **Cameroon** | 555(342 to 832) | 105.45(64.45 to 158.48) | 1151(736 to 1708) | 79.79(51.08 to 117.94) | -0.99(-1.34 to -0.63) | 2404(1688 to 3245) | 474.78(334.48 to 639.56) | 5228(3905 to 6827) | 373.60(280.13 to 485.22) | -0.80(-0.97 to -0.63) | 631(375 to 1237) | 140.77(82.76 to 281.10) | 1431(824 to 2705) | 117.54(67.03 to 228.95) | -0.57(-0.65 to -0.50) |
| **Canada** | 7763(4807 to 11684) | 179.73(111.19 to 270.52) | 11087(7584 to 15780) | 113.71(77.30 to 162.64) | -1.44(-1.51 to -1.37) | 9050(6514 to 12204) | 210.85(151.52 to 284.61) | 15474(11331 to 20726) | 156.66(113.95 to 210.98) | -0.94(-0.99 to -0.90) | 3817(3218 to 4471) | 91.78(77.24 to 107.46) | 5797(4657 to 6957) | 56.04(45.07 to 67.40) | -1.57(-2.01 to -1.12) |
| **Central African Republic** | 123(73 to 190) | 94.18(55.46 to 145.33) | 205(128 to 306) | 85.43(52.99 to 126.82) | -0.44(-0.74 to -0.15) | 790(567 to 1056) | 621.33(448.22 to 826.25) | 1455(1095 to 1875) | 625.92(473.89 to 803.73) | 0.00(-0.04 to 0.04) | 192(99 to 323) | 178.23(91.41 to 316.41) | 373(195 to 641) | 194.94(98.83 to 370.46) | 0.28(0.21 to 0.35) |
| **Chad** | 340(209 to 526) | 97.42(59.82 to 150.54) | 700(445 to 1027) | 105.92(67.12 to 155.13) | 0.09(-0.10 to 0.28) | 2066(1493 to 2706) | 602.92(436.05 to 789.44) | 3865(2897 to 4996) | 600.20(450.32 to 774.98) | -0.08(-0.23 to 0.07) | 498(266 to 1266) | 155.91(81.67 to 409.45) | 1105(585 to 2880) | 193.07(99.54 to 519.60) | 0.69(0.62 to 0.76) |
| **Chile** | 2240(1516 to 3130) | 179.29(121.20 to 250.50) | 5613(3843 to 7858) | 169.37(115.88 to 237.16) | -0.16(-0.29 to -0.03) | 8872(6931 to 11092) | 720.25(563.95 to 898.76) | 14598(11255 to 18570) | 440.21(339.52 to 559.84) | -1.58(-1.61 to -1.55) | 3653(3144 to 4241) | 317.01(272.31 to 367.57) | 3127(2530 to 3824) | 94.12(76.15 to 115.10) | -3.76(-4.80 to -2.72) |
| **China** | 60616(35360 to 95406) | 57.14(33.42 to 89.62) | 228412(136396 to 357906) | 82.63(49.66 to 128.83) | 1.19(1.12 to 1.25) | 211231(136503 to 306809) | 205.15(133.95 to 295.49) | 464489(288858 to 695574) | 169.35(106.12 to 252.34) | -0.64(-0.69 to -0.60) | 24293(16970 to 34583) | 27.88(19.96 to 39.27) | 44631(29205 to 66304) | 17.32(11.53 to 25.36) | -1.56(-1.81 to -1.31) |
| **Colombia** | 7485(4669 to 11208) | 356.90(222.01 to 535.53) | 11162(7659 to 15513) | 161.29(110.81 to 223.94) | -2.52(-2.61 to -2.44) | 21746(16921 to 27632) | 1055.24(822.30 to 1338.23) | 33609(25934 to 42728) | 484.76(374.63 to 615.43) | -2.48(-2.56 to -2.41) | 4398(3672 to 5258) | 225.22(188.45 to 268.22) | 7897(6289 to 9765) | 114.36(91.25 to 141.33) | -2.17(-2.93 to -1.41) |
| **Comoros** | 27(16 to 42) | 114.20(67.17 to 178.81) | 57(36 to 86) | 100.31(63.41 to 149.84) | -0.56(-0.83 to -0.28) | 124(86 to 171) | 523.34(362.22 to 722.59) | 278(202 to 374) | 489.57(355.28 to 658.61) | -0.23(-0.40 to -0.05) | 34(19 to 72) | 166.34(94.05 to 357.75) | 76(42 to 162) | 147.51(81.11 to 318.93) | -0.39(-0.49 to -0.29) |
| **Congo** | 105(64 to 158) | 82.95(50.20 to 125.19) | 246(161 to 357) | 85.79(55.99 to 124.15) | 0.03(-0.17 to 0.23) | 565(406 to 758) | 462.49(333.41 to 618.02) | 1214(926 to 1549) | 438.15(334.62 to 557.89) | -0.27(-0.35 to -0.19) | 149(85 to 247) | 142.57(78.37 to 243.83) | 305(171 to 505) | 128.00(70.28 to 218.95) | -0.35(-0.46 to -0.24) |
| **Cook Islands** | 3(2 to 4) | 163.62(103.73 to 239.83) | 5(3 to 7) | 135.80(91.54 to 192.34) | -0.58(-0.70 to -0.47) | 10(7 to 14) | 683.90(494.12 to 908.15) | 13(10 to 18) | 385.90(282.29 to 510.85) | -1.79(-1.87 to -1.72) | 1(1 to 2) | 82.70(53.04 to 124.08) | 2(1 to 2) | 44.92(26.36 to 71.18) | -1.96(-2.15 to -1.78) |
| **Costa Rica** | 517(312 to 790) | 246.11(148.45 to 375.60) | 1053(706 to 1494) | 151.00(101.55 to 213.85) | -1.56(-1.68 to -1.44) | 1219(880 to 1622) | 583.72(422.28 to 775.16) | 2863(2156 to 3741) | 409.52(309.28 to 533.40) | -1.16(-1.26 to -1.06) | 350(289 to 421) | 171.50(141.52 to 205.79) | 830(669 to 1001) | 119.38(96.51 to 143.95) | -1.21(-1.87 to -0.54) |
| **Coted'Ivoire** | 510(314 to 776) | 114.97(70.23 to 175.26) | 1207(772 to 1756) | 96.60(61.64 to 140.26) | -0.63(-0.88 to -0.38) | 2234(1575 to 3027) | 526.05(372.78 to 709.16) | 5459(4132 to 6995) | 451.27(342.40 to 576.87) | -0.51(-0.81 to -0.21) | 546(317 to 1132) | 152.50(86.95 to 326.21) | 1382(806 to 2779) | 132.73(75.84 to 276.97) | -0.45(-0.52 to -0.38) |
| **Croatia** | 3290(2132 to 4843) | 411.34(266.47 to 606.41) | 3080(2088 to 4358) | 257.29(173.79 to 365.25) | -1.49(-1.55 to -1.43) | 6895(5226 to 8913) | 895.55(682.60 to 1148.51) | 5172(3984 to 6625) | 428.57(328.74 to 551.70) | -2.35(-2.39 to -2.31) | 1573(1297 to 1879) | 223.69(185.48 to 264.98) | 901(707 to 1146) | 71.96(56.16 to 92.12) | -3.57(-3.78 to -3.37) |
| **Cuba** | 2656(1788 to 3835) | 205.99(138.63 to 297.77) | 3859(2637 to 5493) | 160.42(109.50 to 228.89) | -0.79(-0.85 to -0.73) | 8172(6123 to 10501) | 630.12(471.22 to 811.55) | 10876(8263 to 13948) | 448.19(339.17 to 576.65) | -1.10(-1.15 to -1.05) | 1954(1598 to 2365) | 153.83(125.67 to 186.11) | 3272(2632 to 3987) | 129.44(103.98 to 158.05) | -0.55(-1.33 to 0.23) |
| **Cyprus** | 357(216 to 543) | 314.09(189.45 to 479.31) | 490(310 to 728) | 178.33(112.21 to 266.09) | -1.77(-1.88 to -1.66) | 368(246 to 526) | 344.50(229.96 to 493.52) | 729(494 to 1054) | 268.17(180.97 to 390.10) | -0.80(-0.88 to -0.71) | 121(76 to 186) | 167.32(102.52 to 268.65) | 153(100 to 227) | 63.64(40.96 to 96.49) | -3.08(-3.50 to -2.66) |
| **Czechia** | 7815(4739 to 11797) | 419.16(253.81 to 636.05) | 5955(3860 to 8637) | 214.20(137.77 to 312.84) | -2.14(-2.22 to -2.06) | 14227(10820 to 18372) | 773.29(587.88 to 998.02) | 9986(6977 to 13911) | 358.12(246.91 to 505.12) | -2.47(-2.65 to -2.29) | 3242(2722 to 3865) | 179.54(150.54 to 213.81) | 1632(1270 to 2093) | 55.82(42.88 to 72.44) | -3.74(-3.96 to -3.52) |
| **Democratic People's Republic of Korea** | 918(555 to 1423) | 48.31(29.12 to 74.82) | 1711(1060 to 2621) | 42.64(26.46 to 65.17) | -0.41(-0.49 to -0.33) | 2806(1775 to 4092) | 152.12(97.50 to 219.79) | 5328(3505 to 7573) | 133.51(88.23 to 189.28) | -0.46(-0.52 to -0.39) | 360(209 to 563) | 22.17(12.98 to 34.81) | 674(385 to 1097) | 17.51(10.05 to 28.44) | -0.77(-0.85 to -0.69) |
| **Democratic Republic of the Congo** | 1894(1126 to 2868) | 106.38(63.19 to 161.11) | 3489(2248 to 5097) | 85.71(54.86 to 124.80) | -0.79(-1.03 to -0.55) | 8545(6094 to 11471) | 502.80(358.32 to 674.85) | 19195(14705 to 24597) | 483.91(369.76 to 620.15) | -0.18(-0.22 to -0.14) | 2223(1248 to 4116) | 154.27(84.21 to 296.82) | 4821(2567 to 8911) | 142.43(73.56 to 274.11) | -0.26(-0.34 to -0.19) |
| **Denmark** | 3795(2354 to 5775) | 359.84(223.32 to 548.80) | 2704(1782 to 3881) | 177.27(115.17 to 256.46) | -2.30(-2.51 to -2.09) | 4203(2999 to 5682) | 391.35(275.50 to 534.55) | 4078(2975 to 5479) | 264.07(189.11 to 361.08) | -1.27(-1.37 to -1.17) | 1084(890 to 1304) | 98.22(80.23 to 118.57) | 1114(880 to 1363) | 66.88(52.48 to 82.63) | -1.12(-1.81 to -0.42) |
| **Djibouti** | 16(9 to 24) | 106.90(63.63 to 166.01) | 76(48 to 113) | 107.48(67.60 to 159.34) | -0.12(-0.41 to 0.17) | 67(46 to 93) | 467.64(321.26 to 646.30) | 332(237 to 446) | 473.19(338.38 to 635.73) | -0.07(-0.14 to 0.01) | 20(11 to 44) | 168.19(90.78 to 380.14) | 85(49 to 162) | 148.54(83.40 to 290.87) | -0.40(-0.51 to -0.29) |
| **Dominica** | 8(5 to 13) | 108.06(67.45 to 162.44) | 9(6 to 13) | 80.59(51.67 to 119.29) | -0.96(-1.06 to -0.87) | 33(24 to 44) | 427.76(311.57 to 564.50) | 32(24 to 43) | 293.42(214.32 to 390.88) | -1.24(-1.37 to -1.12) | 8(6 to 12) | 106.82(72.67 to 151.66) | 8(5 to 11) | 74.98(49.26 to 110.29) | -1.14(-1.27 to -1.01) |
| **Dominican Republic** | 428(255 to 664) | 95.91(57.07 to 148.52) | 778(478 to 1192) | 63.82(39.21 to 97.73) | -1.32(-1.40 to -1.23) | 1698(1179 to 2341) | 382.36(265.84 to 526.06) | 2897(1962 to 4048) | 237.43(160.97 to 331.55) | -1.57(-1.61 to -1.52) | 704(444 to 1065) | 181.21(113.56 to 273.74) | 801(466 to 1279) | 66.70(38.80 to 106.61) | -3.09(-3.45 to -2.74) |
| **Ecuador** | 1080(691 to 1584) | 172.56(110.22 to 253.22) | 2572(1741 to 3682) | 126.51(85.63 to 181.05) | -1.03(-1.16 to -0.89) | 4020(3045 to 5137) | 648.47(491.67 to 827.86) | 9113(7076 to 11485) | 450.34(349.89 to 567.22) | -1.17(-1.28 to -1.06) | 857(688 to 1073) | 145.27(116.74 to 181.69) | 1992(1560 to 2520) | 100.96(79.29 to 127.39) | -1.28(-1.78 to -0.77) |
| **Egypt** | 4171(2429 to 6540) | 125.69(73.25 to 197.66) | 8388(5049 to 12988) | 101.47(61.55 to 155.73) | -0.73(-1.03 to -0.42) | 9249(5668 to 13875) | 286.08(177.04 to 426.76) | 21185(13210 to 31343) | 266.05(168.52 to 389.11) | -0.26(-0.32 to -0.20) | 1363(861 to 2087) | 49.95(31.20 to 78.28) | 2621(1641 to 3938) | 39.26(24.61 to 59.77) | -0.89(-1.30 to -0.48) |
| **El Salvador** | 1095(677 to 1626) | 306.81(189.93 to 455.79) | 1048(706 to 1471) | 137.29(92.50 to 192.84) | -2.55(-2.64 to -2.45) | 3515(2640 to 4525) | 990.84(744.46 to 1274.93) | 3506(2708 to 4452) | 455.16(351.18 to 578.80) | -2.48(-2.54 to -2.43) | 1198(892 to 1570) | 343.08(255.59 to 450.00) | 1472(1047 to 2052) | 183.40(130.69 to 255.85) | -2.00(-2.56 to -1.44) |
| **Equatorial Guinea** | 16(9 to 24) | 71.00(42.23 to 106.51) | 48(30 to 71) | 89.74(56.89 to 131.66) | 0.70(0.41 to 1.00) | 126(94 to 163) | 583.45(435.90 to 751.83) | 206(151 to 271) | 397.76(291.99 to 522.39) | -1.29(-1.45 to -1.13) | 38(21 to 72) | 196.81(107.10 to 385.90) | 46(27 to 76) | 99.34(55.64 to 165.89) | -2.18(-2.27 to -2.10) |
| **Eritrea** | 98(59 to 150) | 86.09(51.30 to 132.53) | 267(161 to 403) | 89.07(53.92 to 134.02) | -0.10(-0.18 to -0.02) | 586(424 to 784) | 519.21(372.58 to 694.71) | 1476(1052 to 1984) | 492.59(351.06 to 661.42) | -0.24(-0.32 to -0.16) | 193(105 to 403) | 213.90(112.32 to 507.39) | 474(265 to 955) | 195.93(106.13 to 418.49) | -0.28(-0.37 to -0.20) |
| **Estonia** | 654(393 to 1011) | 243.93(146.90 to 377.43) | 537(339 to 802) | 160.53(100.41 to 241.55) | -1.40(-1.89 to -0.91) | 926(618 to 1312) | 347.90(232.63 to 491.26) | 957(642 to 1386) | 281.73(186.66 to 412.27) | -0.71(-0.78 to -0.65) | 179(140 to 227) | 68.40(53.52 to 86.53) | 156(116 to 208) | 42.22(30.91 to 57.21) | -1.61(-2.12 to -1.11) |
| **Eswatini** | 20(12 to 30) | 61.08(37.31 to 91.95) | 36(23 to 52) | 54.94(35.28 to 80.58) | -0.37(-0.53 to -0.21) | 110(80 to 146) | 346.04(252.21 to 457.10) | 186(141 to 238) | 293.11(222.05 to 373.81) | -0.53(-0.64 to -0.42) | 40(25 to 67) | 140.33(85.23 to 241.19) | 67(42 to 107) | 120.25(72.91 to 194.43) | -0.48(-0.58 to -0.38) |
| **Ethiopia** | 2618(1527 to 4114) | 109.62(63.68 to 172.26) | 5958(3601 to 9056) | 121.96(73.62 to 185.40) | 0.31(0.15 to 0.46) | 18491(13309 to 24718) | 785.97(564.67 to 1048.71) | 31400(22087 to 42611) | 647.43(455.45 to 877.87) | -0.61(-0.73 to -0.49) | 3978(2459 to 7118) | 196.54(118.59 to 374.44) | 6348(3853 to 14297) | 141.82(85.39 to 321.63) | -1.06(-1.13 to -0.98) |
| **Fiji** | 30(19 to 46) | 76.70(47.86 to 117.10) | 75(50 to 108) | 77.07(51.47 to 110.79) | 0.04(-0.09 to 0.17) | 131(95 to 173) | 346.59(252.78 to 454.91) | 296(222 to 383) | 316.40(238.52 to 407.23) | -0.26(-0.35 to -0.17) | 18(11 to 29) | 52.18(31.04 to 88.36) | 46(29 to 70) | 55.77(34.01 to 86.95) | 0.12(-0.23 to 0.47) |
| **Finland** | 3362(2095 to 5070) | 357.19(222.83 to 539.63) | 2818(1873 to 4109) | 177.01(116.44 to 261.83) | -2.26(-2.46 to -2.06) | 3888(2747 to 5264) | 412.11(290.06 to 558.95) | 4226(3023 to 5802) | 261.13(183.78 to 364.80) | -1.47(-1.51 to -1.43) | 912(751 to 1095) | 97.23(79.70 to 116.99) | 1023(801 to 1281) | 55.87(43.40 to 71.01) | -1.67(-1.86 to -1.48) |
| **France** | 48419(29858 to 70984) | 449.65(277.82 to 661.13) | 30726(19751 to 45874) | 179.25(113.66 to 270.85) | -2.91(-2.96 to -2.87) | 60493(44605 to 80099) | 549.73(402.98 to 732.14) | 47102(32828 to 65687) | 265.43(181.40 to 376.65) | -2.33(-2.38 to -2.28) | 16780(14012 to 19832) | 148.49(123.31 to 176.14) | 12519(9565 to 15697) | 57.69(43.61 to 73.90) | -3.06(-3.43 to -2.69) |
| **Gabon** | 60(36 to 92) | 84.11(50.22 to 127.85) | 102(65 to 146) | 81.49(52.52 to 116.81) | -0.19(-0.43 to 0.06) | 278(196 to 376) | 395.50(279.91 to 533.78) | 444(337 to 571) | 366.54(277.94 to 470.08) | -0.30(-0.33 to -0.27) | 73(42 to 129) | 114.89(65.93 to 204.56) | 102(61 to 158) | 98.77(57.58 to 155.10) | -0.47(-0.61 to -0.33) |
| **Gambia** | 52(33 to 77) | 126.67(78.92 to 188.12) | 106(68 to 151) | 93.64(60.61 to 134.25) | -1.01(-1.19 to -0.82) | 229(166 to 307) | 575.28(417.26 to 767.14) | 499(382 to 638) | 450.68(344.53 to 575.41) | -0.88(-1.01 to -0.75) | 67(37 to 160) | 185.19(102.32 to 445.73) | 169(93 to 360) | 166.11(90.78 to 355.31) | -0.38(-0.67 to -0.08) |
| **Georgia** | 1024(616 to 1569) | 120.70(72.70 to 185.07) | 1310(901 to 1806) | 161.38(110.81 to 222.63) | 0.97(0.59 to 1.35) | 2280(1500 to 3253) | 279.38(185.72 to 395.40) | 4047(3158 to 5117) | 503.04(392.95 to 635.35) | 1.92(1.84 to 1.99) | 667(529 to 829) | 85.07(67.49 to 105.35) | 713(552 to 897) | 88.27(68.46 to 111.06) | 0.12(-0.96 to 1.22) |
| **Germany** | 60087(37078 to 90937) | 371.73(228.89 to 563.59) | 43407(27177 to 65462) | 184.57(114.28 to 280.78) | -2.22(-2.44 to -2.00) | 75464(54795 to 101167) | 461.82(332.46 to 622.39) | 66680(45167 to 95274) | 278.98(185.80 to 403.49) | -1.64(-1.81 to -1.47) | 15171(12386 to 18670) | 89.77(72.69 to 111.27) | 15695(12020 to 19906) | 56.88(43.13 to 73.28) | -1.44(-1.84 to -1.04) |
| **Ghana** | 701(429 to 1072) | 96.23(58.76 to 147.06) | 1871(1229 to 2743) | 95.17(62.44 to 138.98) | -0.09(-0.35 to 0.16) | 3517(2501 to 4761) | 503.21(359.30 to 678.64) | 8444(6352 to 10860) | 442.89(334.51 to 566.98) | -0.42(-0.49 to -0.35) | 804(476 to 1559) | 131.74(76.10 to 265.31) | 2111(1277 to 3694) | 129.18(76.24 to 239.31) | -0.07(-0.20 to 0.06) |
| **Greece** | 6612(4062 to 9946) | 326.79(200.59 to 492.17) | 3462(2231 to 5014) | 122.30(78.12 to 178.33) | -3.20(-3.54 to -2.87) | 6488(4395 to 9090) | 324.58(219.93 to 454.75) | 5102(3634 to 7017) | 172.95(121.30 to 241.09) | -2.03(-2.16 to -1.90) | 804(581 to 1086) | 41.52(30.17 to 55.75) | 1019(771 to 1313) | 28.59(21.05 to 38.12) | -1.17(-1.43 to -0.92) |
| **Greenland** | 4(2 to 6) | 102.97(63.08 to 157.40) | 8(5 to 11) | 90.39(61.00 to 127.23) | -0.40(-0.53 to -0.27) | 5(3 to 7) | 142.23(96.57 to 200.88) | 16(12 to 21) | 184.63(138.07 to 242.98) | 0.91(0.71 to 1.10) | 2(1 to 3) | 64.17(43.35 to 90.83) | 4(3 to 6) | 60.37(38.38 to 87.56) | -0.08(-0.94 to 0.78) |
| **Grenada** | 11(7 to 16) | 121.47(76.88 to 176.69) | 18(12 to 25) | 123.03(83.44 to 173.42) | 0.04(0.01 to 0.07) | 46(35 to 59) | 509.08(385.80 to 658.44) | 61(47 to 78) | 420.45(325.24 to 538.97) | -0.63(-0.70 to -0.57) | 11(8 to 14) | 109.00(83.65 to 141.74) | 16(13 to 20) | 119.86(96.78 to 147.42) | 0.37(-0.16 to 0.91) |
| **Guam** | 10(6 to 14) | 105.02(69.66 to 151.12) | 19(13 to 28) | 70.26(46.23 to 101.78) | -1.28(-1.31 to -1.26) | 30(22 to 40) | 350.61(256.63 to 462.46) | 56(40 to 75) | 206.53(148.80 to 276.33) | -1.69(-1.72 to -1.66) | 4(3 to 5) | 50.93(35.58 to 73.23) | 6(4 to 9) | 22.65(14.26 to 34.27) | -2.70(-3.72 to -1.67) |
| **Guatemala** | 1378(863 to 2073) | 329.10(206.05 to 495.01) | 2118(1417 to 2990) | 158.74(106.32 to 223.81) | -2.31(-2.38 to -2.23) | 5251(4088 to 6619) | 1290.36(1004.62 to 1624.43) | 8915(7120 to 10997) | 670.32(535.65 to 826.39) | -2.09(-2.11 to -2.08) | 1940(1611 to 2332) | 570.80(470.33 to 698.15) | 4204(3453 to 5102) | 337.88(277.83 to 408.51) | -1.70(-2.87 to -0.52) |
| **Guinea** | 413(248 to 642) | 97.65(58.47 to 151.87) | 591(380 to 857) | 88.78(56.95 to 128.76) | -0.35(-0.46 to -0.25) | 2070(1465 to 2808) | 504.33(357.93 to 682.01) | 3060(2302 to 3957) | 470.26(354.58 to 606.84) | -0.25(-0.49 to -0.01) | 563(302 to 1455) | 149.84(78.93 to 392.60) | 906(488 to 2105) | 151.71(81.00 to 357.17) | 0.05(-0.03 to 0.13) |
| **Guinea-Bissau** | 53(32 to 83) | 111.15(66.30 to 172.61) | 74(47 to 110) | 90.81(56.88 to 134.09) | -0.73(-1.01 to -0.45) | 312(226 to 419) | 673.14(487.78 to 899.72) | 423(313 to 551) | 534.30(397.19 to 694.04) | -0.76(-0.85 to -0.67) | 91(49 to 174) | 221.87(118.08 to 444.72) | 131(74 to 251) | 196.80(108.73 to 399.34) | -0.38(-0.46 to -0.30) |
| **Guyana** | 69(45 to 99) | 149.90(97.21 to 214.26) | 90(59 to 130) | 109.83(72.40 to 158.30) | -0.98(-1.14 to -0.83) | 386(306 to 485) | 848.98(671.40 to 1066.74) | 411(322 to 514) | 504.48(395.54 to 628.94) | -1.67(-1.73 to -1.61) | 100(80 to 123) | 238.09(190.84 to 292.07) | 110(84 to 142) | 147.64(111.84 to 190.07) | -1.43(-2.52 to -0.33) |
| **Haiti** | 688(404 to 1064) | 168.59(98.55 to 261.68) | 886(546 to 1330) | 105.30(64.70 to 158.07) | -1.51(-1.57 to -1.44) | 3740(2645 to 5032) | 935.13(660.31 to 1258.96) | 5352(3927 to 7082) | 643.42(471.20 to 852.71) | -1.21(-1.27 to -1.15) | 905(563 to 1408) | 257.01(156.16 to 415.56) | 1438(883 to 2267) | 194.90(117.66 to 314.64) | -0.89(-1.00 to -0.77) |
| **Honduras** | 859(524 to 1302) | 355.88(216.96 to 540.42) | 1601(1039 to 2293) | 210.14(136.27 to 300.68) | -1.67(-1.81 to -1.53) | 3485(2556 to 4606) | 1475.82(1082.39 to 1950.37) | 7114(5412 to 9089) | 949.11(722.86 to 1211.06) | -1.41(-1.47 to -1.35) | 780(536 to 1138) | 348.85(237.48 to 516.09) | 2104(1185 to 3297) | 301.19(167.98 to 474.05) | -0.45(-0.66 to -0.24) |
| **Hungary** | 7981(5039 to 11672) | 398.62(251.84 to 583.64) | 5619(3766 to 7912) | 215.85(144.28 to 305.70) | -1.96(-2.05 to -1.86) | 14491(11140 to 18658) | 739.91(569.61 to 950.40) | 10366(7786 to 13636) | 394.03(293.31 to 522.13) | -2.01(-2.06 to -1.95) | 3606(3062 to 4237) | 193.19(164.19 to 226.50) | 1952(1539 to 2453) | 72.57(57.02 to 91.58) | -3.07(-3.39 to -2.75) |
| **Iceland** | 104(63 to 161) | 283.60(171.66 to 436.94) | 93(55 to 151) | 124.86(73.28 to 202.67) | -2.62(-2.87 to -2.37) | 89(60 to 126) | 238.78(160.49 to 341.45) | 112(70 to 169) | 148.64(92.31 to 224.76) | -1.54(-1.59 to -1.50) | 21(17 to 27) | 56.12(44.33 to 70.32) | 30(23 to 39) | 37.49(27.95 to 48.64) | -1.27(-1.86 to -0.68) |
| **India** | 101974(61789 to 154452) | 192.59(116.44 to 292.59) | 242011(145579 to 371152) | 166.17(100.13 to 254.15) | -0.48(-0.58 to -0.39) | 552758(411601 to 715911) | 1081.97(809.16 to 1397.24) | 799401(567577 to 1071327) | 561.45(400.10 to 750.80) | -2.13(-2.25 to -2.01) | 145144(109764 to 195612) | 312.50(234.33 to 424.06) | 189426(139582 to 254719) | 142.58(104.00 to 191.82) | -2.54(-3.02 to -2.06) |
| **Indonesia** | 21439(12932 to 32866) | 190.37(114.35 to 292.51) | 57822(37572 to 82974) | 201.22(130.27 to 288.97) | 0.21(0.16 to 0.26) | 113387(80854 to 153463) | 1054.26(753.69 to 1422.92) | 247242(187166 to 319758) | 906.38(688.62 to 1166.46) | -0.48(-0.54 to -0.42) | 41623(27289 to 68429) | 432.65(280.99 to 713.81) | 86484(57806 to 112711) | 374.07(253.33 to 488.64) | -0.47(-0.64 to -0.31) |
| **Iran (Islamic Republic of)** | 2990(1709 to 4791) | 83.75(47.87 to 134.15) | 9406(5505 to 14919) | 97.86(57.47 to 154.88) | 0.48(0.43 to 0.53) | 8773(5318 to 13344) | 252.87(154.89 to 381.26) | 21320(12991 to 32376) | 224.20(137.72 to 338.15) | -0.40(-0.49 to -0.31) | 770(484 to 1181) | 25.15(16.31 to 37.77) | 1920(1223 to 2894) | 21.06(13.62 to 31.29) | -0.61(-0.78 to -0.45) |
| **Iraq** | 1247(722 to 2030) | 135.31(78.38 to 220.31) | 3072(1823 to 4882) | 107.42(64.22 to 169.55) | -0.75(-0.91 to -0.60) | 2323(1396 to 3555) | 253.02(152.34 to 386.65) | 7502(4657 to 11485) | 267.15(167.49 to 404.58) | 0.13(-0.03 to 0.30) | 234(128 to 409) | 25.81(14.16 to 45.40) | 635(352 to 1068) | 23.93(13.41 to 40.74) | -0.26(-0.36 to -0.16) |
| **Ireland** | 1902(1167 to 2886) | 343.52(210.50 to 521.47) | 1867(1210 to 2736) | 184.76(118.99 to 272.01) | -1.96(-2.10 to -1.82) | 2386(1720 to 3205) | 433.88(310.49 to 586.07) | 2570(1774 to 3568) | 252.43(172.67 to 353.26) | -1.75(-1.81 to -1.69) | 648(540 to 772) | 123.18(102.46 to 146.73) | 526(401 to 673) | 49.72(37.77 to 64.15) | -2.81(-3.30 to -2.32) |
| **Israel** | 2157(1337 to 3236) | 331.53(205.79 to 498.24) | 2368(1481 to 3593) | 152.13(94.59 to 232.15) | -2.48(-2.63 to -2.34) | 2488(1775 to 3370) | 393.73(280.35 to 534.14) | 3664(2459 to 5229) | 232.39(154.71 to 334.30) | -1.68(-1.78 to -1.58) | 551(447 to 676) | 92.20(74.74 to 112.79) | 918(694 to 1164) | 53.92(40.56 to 69.05) | -1.63(-1.98 to -1.29) |
| **Italy** | 41884(26360 to 62471) | 351.93(221.81 to 526.14) | 42625(26703 to 63916) | 243.53(151.46 to 368.53) | -1.18(-1.33 to -1.04) | 81381(58745 to 107351) | 687.64(495.09 to 908.27) | 70157(48052 to 96769) | 387.66(261.76 to 539.60) | -1.84(-1.90 to -1.79) | 13269(10971 to 15999) | 113.55(93.45 to 137.05) | 11654(9119 to 14355) | 56.29(43.62 to 70.90) | -2.26(-2.66 to -1.85) |
| **Jamaica** | 271(170 to 409) | 120.39(75.58 to 181.33) | 390(258 to 555) | 100.60(66.62 to 143.15) | -0.58(-0.66 to -0.50) | 908(635 to 1239) | 401.08(280.41 to 547.67) | 1343(1002 to 1735) | 345.54(258.05 to 446.11) | -0.49(-0.56 to -0.41) | 230(184 to 286) | 99.25(79.28 to 123.58) | 358(273 to 465) | 90.73(69.32 to 118.03) | -0.20(-1.38 to 1.00) |
| **Japan** | 36857(22116 to 56635) | 167.86(100.90 to 257.64) | 79627(48250 to 123748) | 186.93(111.66 to 293.93) | 0.33(0.24 to 0.43) | 61748(40434 to 89333) | 287.35(189.55 to 413.36) | 104769(68952 to 151618) | 231.21(146.81 to 344.05) | -0.72(-0.80 to -0.65) | 9515(7605 to 11937) | 45.99(36.78 to 57.30) | 17379(12663 to 22391) | 29.76(21.38 to 40.14) | -1.44(-1.90 to -0.98) |
| **Jordan** | 243(141 to 391) | 160.16(93.10 to 258.15) | 1115(661 to 1714) | 127.75(76.40 to 195.97) | -0.68(-0.92 to -0.44) | 438(271 to 669) | 298.40(187.18 to 450.46) | 2433(1552 to 3583) | 286.45(184.85 to 418.29) | -0.18(-0.36 to 0.00) | 35(20 to 57) | 24.75(14.18 to 39.57) | 187(103 to 307) | 22.88(12.88 to 36.99) | -0.30(-0.57 to -0.03) |
| **Kazakhstan** | 2722(1810 to 3858) | 161.77(106.58 to 231.09) | 2356(1535 to 3512) | 97.59(63.34 to 145.32) | -1.65(-1.81 to -1.48) | 12087(9608 to 14956) | 760.45(605.44 to 937.88) | 7288(5192 to 9882) | 311.91(223.76 to 420.15) | -2.87(-3.02 to -2.72) | 2049(1606 to 2574) | 133.41(104.64 to 167.38) | 1235(928 to 1610) | 56.76(42.94 to 73.42) | -2.79(-3.53 to -2.03) |
| **Kenya** | 1232(721 to 1943) | 127.74(74.55 to 201.68) | 3750(2315 to 5636) | 137.64(84.80 to 206.66) | 0.24(0.01 to 0.46) | 6644(4537 to 9252) | 699.11(477.02 to 972.16) | 18913(13659 to 25181) | 706.47(510.65 to 938.59) | 0.03(-0.20 to 0.27) | 1485(860 to 3448) | 174.46(99.81 to 412.04) | 3856(2599 to 7348) | 170.69(112.53 to 344.54) | -0.08(-0.15 to 0.00) |
| **Kiribati** | 4(2 to 6) | 83.08(50.00 to 128.31) | 5(3 to 8) | 61.87(39.32 to 90.65) | -0.94(-0.99 to -0.89) | 21(15 to 28) | 478.72(343.01 to 637.51) | 29(21 to 38) | 347.36(258.56 to 450.70) | -1.03(-1.09 to -0.97) | 3(2 to 4) | 65.34(39.42 to 107.71) | 4(2 to 6) | 52.07(30.79 to 87.91) | -0.74(-0.85 to -0.64) |
| **Kuwait** | 156(95 to 243) | 253.66(154.98 to 392.34) | 470(284 to 726) | 159.04(97.41 to 243.15) | -1.47(-1.70 to -1.25) | 263(175 to 375) | 448.70(303.10 to 631.92) | 837(537 to 1258) | 295.51(193.54 to 436.85) | -1.33(-1.45 to -1.21) | 21(12 to 32) | 36.54(22.23 to 54.24) | 79(49 to 121) | 29.22(18.68 to 43.58) | -0.63(-2.11 to 0.87) |
| **Kyrgyzstan** | 479(294 to 729) | 121.67(74.23 to 186.98) | 491(301 to 756) | 77.95(47.86 to 119.46) | -1.43(-1.55 to -1.31) | 1371(963 to 1873) | 359.34(253.96 to 488.18) | 1865(1259 to 2559) | 302.13(206.55 to 411.60) | -0.61(-0.77 to -0.45) | 203(153 to 265) | 55.69(42.10 to 72.14) | 217(153 to 303) | 37.86(27.20 to 52.38) | -1.50(-2.53 to -0.45) |
| **Lao People's Democratic Republic** | 248(148 to 385) | 98.37(58.46 to 152.28) | 466(299 to 681) | 87.57(56.12 to 127.88) | -0.41(-0.59 to -0.22) | 732(500 to 1006) | 302.41(206.78 to 415.48) | 1407(1010 to 1870) | 272.28(195.95 to 360.91) | -0.36(-0.45 to -0.28) | 238(131 to 480) | 107.24(58.30 to 216.45) | 398(223 to 725) | 85.42(47.21 to 155.89) | -0.74(-0.79 to -0.70) |
| **Latvia** | 1324(810 to 2022) | 285.15(174.64 to 436.28) | 690(427 to 1031) | 138.76(85.12 to 208.73) | -2.38(-2.76 to -1.99) | 1996(1403 to 2747) | 436.66(308.54 to 597.71) | 1368(912 to 1966) | 271.15(178.22 to 393.86) | -1.55(-1.62 to -1.48) | 391(313 to 487) | 85.59(68.56 to 106.45) | 297(230 to 379) | 54.03(41.42 to 69.52) | -1.45(-1.85 to -1.04) |
| **Lebanon** | 424(243 to 681) | 149.26(85.56 to 239.49) | 832(487 to 1310) | 119.14(69.40 to 188.24) | -0.71(-0.89 to -0.53) | 745(440 to 1162) | 267.05(159.44 to 413.74) | 1853(1145 to 2834) | 261.90(160.96 to 402.79) | -0.11(-0.22 to 0.01) | 154(96 to 240) | 63.13(38.72 to 99.52) | 272(171 to 411) | 35.81(22.53 to 54.37) | -1.82(-1.96 to -1.67) |
| **Lesotho** | 61(37 to 91) | 58.60(35.81 to 87.68) | 80(51 to 117) | 59.07(38.06 to 86.19) | -0.01(-0.16 to 0.15) | 412(302 to 538) | 401.35(294.79 to 523.89) | 483(370 to 617) | 362.58(278.06 to 461.76) | -0.31(-0.41 to -0.20) | 142(80 to 291) | 147.88(82.48 to 309.44) | 190(115 to 299) | 159.88(95.09 to 254.65) | 0.27(0.14 to 0.41) |
| **Liberia** | 199(119 to 308) | 135.62(80.74 to 209.89) | 249(156 to 367) | 114.21(71.78 to 168.37) | -0.65(-0.97 to -0.32) | 763(505 to 1076) | 544.00(360.72 to 765.44) | 1101(805 to 1462) | 525.72(384.30 to 698.64) | -0.12(-0.28 to 0.04) | 182(102 to 419) | 143.22(79.06 to 335.48) | 266(143 to 669) | 138.03(73.09 to 353.21) | -0.13(-0.34 to 0.09) |
| **Libya** | 382(222 to 610) | 170.60(99.19 to 272.76) | 582(338 to 910) | 101.01(59.15 to 157.03) | -1.70(-1.85 to -1.56) | 573(342 to 888) | 259.70(156.01 to 401.24) | 1503(933 to 2223) | 265.61(166.35 to 389.38) | 0.03(-0.10 to 0.15) | 61(31 to 109) | 28.71(14.75 to 51.63) | 144(76 to 244) | 26.39(13.93 to 45.01) | -0.31(-0.48 to -0.14) |
| **Lithuania** | 1747(1056 to 2664) | 300.28(181.22 to 459.35) | 1243(811 to 1805) | 171.26(111.14 to 250.24) | -1.86(-2.18 to -1.53) | 2906(2056 to 3947) | 505.13(359.52 to 682.51) | 2571(1870 to 3489) | 347.65(250.29 to 476.10) | -1.21(-1.26 to -1.15) | 582(468 to 716) | 101.81(82.07 to 124.86) | 584(465 to 724) | 72.65(57.44 to 90.80) | -1.12(-1.76 to -0.48) |
| **Luxembourg** | 290(176 to 441) | 404.97(245.11 to 617.22) | 262(164 to 389) | 200.77(125.07 to 299.01) | -2.23(-2.40 to -2.05) | 403(297 to 536) | 563.46(414.77 to 749.66) | 396(267 to 571) | 299.66(200.58 to 434.40) | -2.03(-2.14 to -1.92) | 81(66 to 98) | 116.33(94.38 to 140.78) | 82(62 to 106) | 56.87(42.59 to 74.81) | -2.32(-2.48 to -2.15) |
| **Madagascar** | 584(346 to 900) | 97.06(57.46 to 149.63) | 1175(740 to 1773) | 94.75(59.50 to 142.76) | -0.24(-0.56 to 0.08) | 2574(1751 to 3610) | 430.60(292.61 to 603.02) | 5674(4102 to 7499) | 461.39(332.49 to 610.82) | 0.10(0.06 to 0.14) | 906(491 to 2218) | 172.73(91.42 to 432.27) | 1570(846 to 3918) | 156.66(81.71 to 406.97) | -0.32(-0.42 to -0.23) |
| **Malawi** | 437(261 to 679) | 95.01(56.72 to 147.62) | 841(533 to 1245) | 97.79(61.80 to 144.89) | -0.03(-0.30 to 0.23) | 2035(1421 to 2768) | 444.40(310.11 to 605.13) | 4113(3050 to 5416) | 479.73(354.96 to 631.75) | 0.23(0.16 to 0.31) | 638(372 to 1351) | 163.97(93.28 to 354.72) | 1127(663 to 2296) | 151.26(86.94 to 316.19) | -0.25(-0.41 to -0.09) |
| **Malaysia** | 1937(1210 to 2897) | 183.75(114.46 to 274.96) | 6489(4388 to 8992) | 182.66(123.40 to 253.00) | -0.01(-0.17 to 0.16) | 4957(3640 to 6540) | 482.98(355.30 to 636.32) | 15577(11942 to 19818) | 457.05(351.43 to 580.19) | -0.19(-0.23 to -0.14) | 1513(965 to 2308) | 150.43(96.08 to 229.38) | 4485(2881 to 6686) | 141.95(90.67 to 212.44) | -0.40(-0.65 to -0.15) |
| **Maldives** | 18(11 to 28) | 189.02(115.50 to 288.73) | 43(29 to 62) | 126.29(83.76 to 178.82) | -1.32(-1.42 to -1.22) | 40(27 to 55) | 447.72(309.57 to 618.98) | 96(69 to 129) | 282.98(206.99 to 377.12) | -1.51(-1.66 to -1.36) | 12(7 to 20) | 158.74(93.01 to 261.74) | 22(14 to 33) | 69.44(45.08 to 102.07) | -2.70(-3.01 to -2.39) |
| **Mali** | 468(282 to 718) | 96.73(58.14 to 148.63) | 1064(674 to 1561) | 102.72(64.76 to 150.71) | 0.16(0.01 to 0.31) | 2548(1812 to 3408) | 552.95(395.18 to 736.42) | 5184(3824 to 6810) | 519.57(385.13 to 680.32) | -0.30(-0.38 to -0.22) | 821(439 to 1955) | 205.83(108.70 to 494.77) | 1725(888 to 4283) | 197.81(100.05 to 502.40) | -0.13(-0.24 to -0.02) |
| **Malta** | 214(131 to 325) | 383.61(235.29 to 581.10) | 224(144 to 328) | 175.86(111.87 to 259.75) | -2.50(-2.57 to -2.43) | 307(225 to 404) | 557.22(408.48 to 733.78) | 346(238 to 486) | 268.78(181.87 to 382.18) | -2.34(-2.41 to -2.28) | 74(60 to 90) | 141.14(115.15 to 170.72) | 96(74 to 120) | 68.59(52.72 to 87.04) | -2.52(-2.71 to -2.33) |
| **Marshall Islands** | 2(1 to 3) | 97.86(59.96 to 146.55) | 3(2 to 5) | 81.49(52.68 to 120.81) | -0.58(-0.65 to -0.52) | 10(7 to 13) | 523.47(374.37 to 694.87) | 15(11 to 19) | 394.42(293.14 to 510.72) | -0.90(-1.00 to -0.79) | 1(1 to 2) | 81.06(49.67 to 132.50) | 2(1 to 3) | 63.55(37.85 to 101.61) | -0.78(-0.90 to -0.65) |
| **Mauritania** | 129(78 to 202) | 106.24(63.91 to 165.01) | 246(159 to 357) | 94.11(60.92 to 136.42) | -0.49(-0.83 to -0.15) | 589(414 to 802) | 495.69(349.25 to 674.55) | 1008(747 to 1315) | 396.50(294.91 to 514.80) | -0.78(-0.90 to -0.65) | 157(87 to 341) | 143.38(77.71 to 316.77) | 281(151 to 676) | 121.42(64.25 to 295.21) | -0.54(-0.62 to -0.47) |
| **Mauritius** | 75(45 to 117) | 79.28(48.51 to 123.59) | 138(84 to 211) | 54.29(33.37 to 82.42) | -1.26(-1.46 to -1.05) | 126(85 to 180) | 138.58(94.59 to 194.97) | 296(188 to 437) | 117.99(75.96 to 172.85) | -0.57(-0.62 to -0.52) | 15(10 to 21) | 17.41(12.00 to 24.42) | 57(43 to 74) | 24.71(18.97 to 31.75) | 0.08(-0.44 to 0.60) |
| **Mexico** | 16325(10386 to 23788) | 323.25(205.34 to 472.17) | 30627(20152 to 43812) | 196.21(129.19 to 280.56) | -1.60(-1.66 to -1.54) | 70430(55246 to 87418) | 1427.39(1120.56 to 1769.88) | 121169(94913 to 150274) | 782.42(613.61 to 969.18) | -1.93(-1.97 to -1.88) | 15164(13370 to 17204) | 337.20(299.01 to 379.72) | 29412(25237 to 34067) | 196.64(169.03 to 227.24) | -1.72(-2.29 to -1.15) |
| **Micronesia (Federated States of)** | 6(4 to 9) | 95.39(58.28 to 144.11) | 7(4 to 10) | 72.87(47.27 to 106.55) | -0.85(-0.92 to -0.77) | 30(21 to 40) | 482.65(346.72 to 644.69) | 29(21 to 38) | 329.32(244.76 to 426.69) | -1.22(-1.28 to -1.16) | 4(3 to 7) | 73.32(44.88 to 129.77) | 4(2 to 6) | 49.17(29.75 to 77.23) | -1.28(-1.34 to -1.22) |
| **Monaco** | 32(20 to 48) | 347.35(211.21 to 521.78) | 20(12 to 30) | 164.33(100.72 to 252.56) | -2.38(-2.50 to -2.25) | 35(24 to 49) | 367.42(247.75 to 516.70) | 30(21 to 44) | 242.02(161.29 to 355.64) | -1.35(-1.40 to -1.29) | 7(4 to 10) | 64.36(40.29 to 99.82) | 6(4 to 10) | 44.60(26.75 to 70.97) | -1.18(-1.23 to -1.14) |
| **Mongolia** | 217(132 to 326) | 172.02(104.89 to 258.32) | 180(110 to 282) | 65.71(40.11 to 102.62) | -3.07(-3.19 to -2.95) | 1179(877 to 1529) | 945.00(702.84 to 1224.57) | 680(454 to 949) | 254.69(172.00 to 353.21) | -4.21(-4.31 to -4.10) | 142(97 to 201) | 116.57(79.75 to 165.84) | 110(74 to 160) | 45.91(30.48 to 67.00) | -3.00(-3.22 to -2.78) |
| **Montenegro** | 212(123 to 329) | 269.55(156.69 to 419.65) | 234(143 to 362) | 169.09(103.59 to 260.80) | -1.51(-1.74 to -1.28) | 258(157 to 396) | 330.31(203.06 to 504.56) | 408(259 to 617) | 297.89(189.52 to 448.29) | -0.40(-0.48 to -0.31) | 29(19 to 43) | 37.86(25.03 to 55.59) | 45(28 to 68) | 35.02(22.28 to 52.36) | -0.24(-0.41 to -0.08) |
| **Morocco** | 2420(1395 to 3843) | 137.00(78.96 to 217.61) | 4476(2676 to 6832) | 100.44(60.40 to 152.95) | -0.99(-1.10 to -0.88) | 5588(3454 to 8423) | 319.34(197.89 to 480.65) | 12307(7743 to 18128) | 281.00(178.64 to 411.37) | -0.45(-0.51 to -0.38) | 539(298 to 957) | 31.82(17.62 to 57.46) | 1124(637 to 1907) | 26.86(15.31 to 46.33) | -0.56(-0.68 to -0.44) |
| **Mozambique** | 628(377 to 971) | 90.26(54.03 to 139.71) | 1412(892 to 2067) | 109.39(68.66 to 160.20) | 0.55(0.37 to 0.74) | 3617(2578 to 4875) | 523.39(372.16 to 705.25) | 7138(5278 to 9353) | 559.51(412.29 to 734.01) | 0.21(0.11 to 0.31) | 1078(604 to 2512) | 177.90(97.88 to 421.71) | 2036(1182 to 3887) | 183.91(104.34 to 360.83) | 0.11(0.03 to 0.20) |
| **Myanmar** | 2575(1544 to 3997) | 89.47(53.44 to 138.82) | 4482(2923 to 6393) | 72.91(47.57 to 103.90) | -0.72(-1.00 to -0.43) | 7913(5489 to 10816) | 282.57(196.23 to 386.33) | 13177(9745 to 17179) | 216.99(160.86 to 282.31) | -0.89(-0.96 to -0.83) | 2689(1413 to 5430) | 101.57(52.72 to 208.52) | 3845(2229 to 6703) | 68.02(39.34 to 119.07) | -1.29(-1.35 to -1.23) |
| **Namibia** | 65(40 to 98) | 79.01(48.38 to 118.50) | 125(79 to 185) | 77.34(49.08 to 114.02) | -0.11(-0.24 to 0.03) | 375(275 to 497) | 464.00(340.76 to 613.88) | 610(463 to 784) | 383.72(291.58 to 492.02) | -0.60(-0.70 to -0.50) | 124(73 to 233) | 179.39(101.86 to 353.09) | 204(128 to 319) | 144.56(89.89 to 230.29) | -0.69(-0.80 to -0.58) |
| **Nauru** | 0(0 to 1) | 90.03(53.52 to 137.95) | 0(0 to 0) | 39.21(24.45 to 59.07) | -2.62(-2.73 to -2.50) | 2(1 to 2) | 317.13(220.60 to 437.23) | 1(1 to 2) | 181.43(132.56 to 237.98) | -1.78(-1.82 to -1.74) | 0(0 to 0) | 68.65(35.71 to 113.05) | 0(0 to 0) | 42.36(19.18 to 70.87) | -1.53(-1.62 to -1.45) |
| **Nepal** | 1813(1085 to 2745) | 166.71(99.48 to 252.59) | 3228(2081 to 4687) | 113.48(73.23 to 164.40) | -1.20(-1.30 to -1.11) | 11552(8486 to 15102) | 1103.10(811.58 to 1438.49) | 13107(9884 to 16957) | 473.73(357.49 to 612.05) | -2.69(-2.74 to -2.64) | 2872(1599 to 6161) | 305.12(166.48 to 659.48) | 3138(1875 to 5799) | 124.92(74.04 to 231.94) | -2.84(-2.94 to -2.74) |
| **Netherlands** | 9361(5754 to 14135) | 360.03(220.87 to 545.06) | 8808(5696 to 12822) | 193.40(124.25 to 283.26) | -1.97(-2.09 to -1.86) | 10308(7173 to 14298) | 394.29(272.39 to 550.10) | 12472(8667 to 17697) | 271.45(186.52 to 389.76) | -1.21(-1.25 to -1.18) | 3182(2609 to 3818) | 121.23(98.96 to 145.86) | 3288(2562 to 4104) | 66.69(51.68 to 83.91) | -1.91(-2.56 to -1.26) |
| **New Zealand** | 1063(659 to 1603) | 202.64(125.62 to 305.86) | 1688(1062 to 2506) | 151.19(94.75 to 224.95) | -0.93(-0.98 to -0.87) | 2391(1704 to 3224) | 458.65(326.24 to 619.28) | 3435(2423 to 4639) | 305.41(214.19 to 414.39) | -1.29(-1.36 to -1.22) | 538(440 to 648) | 105.68(86.28 to 127.29) | 811(641 to 995) | 69.92(55.24 to 86.15) | -1.06(-2.13 to 0.01) |
| **Nicaragua** | 626(393 to 941) | 351.88(221.01 to 528.41) | 881(598 to 1229) | 151.33(102.72 to 210.83) | -2.67(-2.81 to -2.53) | 1825(1399 to 2323) | 1045.48(802.59 to 1329.21) | 2920(2256 to 3692) | 505.31(391.46 to 637.16) | -2.31(-2.36 to -2.27) | 364(274 to 475) | 218.46(164.47 to 285.89) | 733(537 to 981) | 132.93(96.90 to 178.42) | -1.64(-1.83 to -1.44) |
| **Niger** | 386(230 to 583) | 116.80(69.37 to 177.06) | 1011(630 to 1478) | 102.08(63.13 to 149.36) | -0.49(-0.61 to -0.36) | 1901(1365 to 2536) | 597.26(429.96 to 795.61) | 5421(3972 to 7082) | 569.60(419.19 to 742.33) | -0.24(-0.32 to -0.16) | 498(261 to 1346) | 182.85(93.61 to 504.23) | 1629(771 to 5336) | 202.22(93.54 to 666.78) | 0.34(0.31 to 0.36) |
| **Nigeria** | 6508(3833 to 10266) | 119.38(70.18 to 188.33) | 12697(7679 to 19273) | 125.70(76.00 to 190.67) | 0.17(0.02 to 0.32) | 34470(23504 to 47896) | 649.38(444.17 to 899.63) | 60184(42141 to 81604) | 616.37(433.95 to 832.48) | -0.16(-0.25 to -0.07) | 6622(4337 to 13323) | 139.18(90.28 to 287.16) | 11584(7650 to 22483) | 131.83(86.81 to 260.23) | -0.17(-0.22 to -0.12) |
| **Niue** | 0(0 to 0) | 87.23(56.38 to 129.65) | 0(0 to 0) | 74.18(49.06 to 107.28) | -0.53(-0.61 to -0.45) | 1(1 to 1) | 364.09(264.88 to 487.40) | 1(1 to 1) | 253.90(186.16 to 334.44) | -1.15(-1.22 to -1.07) | 0(0 to 0) | 50.39(32.59 to 75.72) | 0(0 to 0) | 35.40(22.08 to 53.89) | -1.12(-1.15 to -1.09) |
| **North Macedonia** | 919(573 to 1394) | 391.68(244.21 to 594.68) | 870(570 to 1272) | 188.98(124.17 to 275.24) | -2.33(-2.39 to -2.28) | 1504(1094 to 2002) | 655.88(479.72 to 868.52) | 1653(1166 to 2272) | 370.82(263.89 to 506.05) | -1.84(-1.89 to -1.80) | 247(181 to 325) | 114.10(83.71 to 150.12) | 244(169 to 339) | 63.16(44.25 to 87.50) | -1.87(-2.14 to -1.59) |
| **Northern Mariana Islands** | 1(1 to 2) | 83.15(49.90 to 128.55) | 4(2 to 6) | 58.54(36.11 to 89.94) | -1.11(-1.34 to -0.87) | 3(2 to 4) | 202.03(130.95 to 291.86) | 10(7 to 15) | 167.74(111.01 to 237.59) | -0.67(-1.04 to -0.29) | 0(0 to 0) | 15.84(8.50 to 26.33) | 1(0 to 1) | 13.61(6.98 to 22.69) | -0.52(-0.81 to -0.23) |
| **Norway** | 2573(1612 to 3860) | 282.18(176.25 to 425.03) | 2223(1387 to 3353) | 172.12(106.67 to 261.36) | -1.55(-1.63 to -1.48) | 5092(3557 to 6969) | 543.99(374.61 to 753.17) | 3684(2464 to 5272) | 279.10(183.56 to 404.39) | -2.15(-2.24 to -2.06) | 1180(1006 to 1381) | 121.17(102.58 to 142.96) | 885(713 to 1058) | 61.25(49.14 to 74.25) | -2.00(-2.39 to -1.60) |
| **Oman** | 121(70 to 191) | 161.42(93.63 to 254.14) | 246(143 to 389) | 114.59(66.90 to 179.70) | -1.09(-1.16 to -1.02) | 230(140 to 350) | 309.13(189.43 to 468.77) | 504(301 to 771) | 243.44(148.23 to 368.35) | -0.81(-0.91 to -0.71) | 23(13 to 38) | 31.75(18.32 to 54.29) | 46(26 to 76) | 24.15(13.54 to 39.33) | -0.86(-1.07 to -0.65) |
| **Pakistan** | 10041(5971 to 15569) | 149.08(88.57 to 231.09) | 9523(5772 to 14548) | 69.72(42.30 to 106.00) | -2.43(-2.49 to -2.37) | 57824(40860 to 78096) | 881.31(623.86 to 1189.02) | 44548(31360 to 59688) | 336.15(237.94 to 448.81) | -3.08(-3.15 to -3.01) | 12373(6834 to 26259) | 203.50(110.52 to 436.21) | 11480(7697 to 17672) | 97.53(65.01 to 151.29) | -2.34(-2.45 to -2.22) |
| **Palau** | 1(1 to 2) | 102.65(65.52 to 151.00) | 2(2 to 3) | 83.53(55.34 to 120.65) | -0.66(-0.74 to -0.58) | 5(4 to 7) | 423.72(307.47 to 558.34) | 8(6 to 10) | 292.68(216.82 to 384.26) | -1.17(-1.28 to -1.07) | 1(0 to 1) | 57.22(36.25 to 83.55) | 1(1 to 1) | 35.42(21.97 to 53.14) | -1.52(-1.63 to -1.42) |
| **Palestine** | 148(87 to 231) | 135.70(79.74 to 212.35) | 352(207 to 548) | 115.84(68.80 to 179.28) | -0.48(-0.66 to -0.31) | 320(199 to 484) | 297.50(186.16 to 447.09) | 870(540 to 1319) | 291.55(182.71 to 438.02) | -0.12(-0.28 to 0.04) | 78(44 to 142) | 79.67(44.22 to 147.18) | 112(71 to 166) | 42.31(26.90 to 62.56) | -2.05(-2.30 to -1.80) |
| **Panama** | 427(256 to 647) | 237.95(143.01 to 359.92) | 691(442 to 1011) | 126.57(80.94 to 185.38) | -2.00(-2.14 to -1.87) | 1214(883 to 1608) | 681.20(495.68 to 901.91) | 1923(1329 to 2661) | 351.24(242.52 to 486.38) | -2.14(-2.19 to -2.09) | 355(290 to 430) | 205.26(167.89 to 249.17) | 646(490 to 812) | 115.93(88.11 to 145.88) | -1.89(-2.78 to -0.99) |
| **Papua New Guinea** | 153(91 to 237) | 70.16(41.46 to 108.93) | 349(215 to 532) | 61.79(37.73 to 94.08) | -0.40(-0.49 to -0.30) | 716(481 to 998) | 347.03(234.99 to 479.52) | 1600(1135 to 2156) | 293.22(208.31 to 393.67) | -0.54(-0.64 to -0.44) | 77(44 to 153) | 42.11(23.59 to 88.40) | 179(97 to 358) | 36.74(19.53 to 77.03) | -0.44(-0.51 to -0.37) |
| **Paraguay** | 550(344 to 815) | 205.27(128.34 to 303.76) | 1105(711 to 1601) | 153.85(99.04 to 222.55) | -0.93(-0.99 to -0.88) | 2327(1669 to 3118) | 875.70(628.30 to 1172.58) | 3714(2696 to 4875) | 519.02(377.05 to 681.19) | -1.67(-1.72 to -1.62) | 744(549 to 1036) | 290.00(214.03 to 403.77) | 2336(1639 to 3239) | 342.40(240.08 to 474.62) | 0.62(0.27 to 0.97) |
| **Peru** | 2291(1440 to 3376) | 161.58(101.21 to 238.38) | 4480(2893 to 6540) | 110.16(71.20 to 160.69) | -1.23(-1.35 to -1.11) | 9322(6969 to 12137) | 663.04(495.83 to 862.98) | 13994(10173 to 18652) | 343.55(249.91 to 457.63) | -2.12(-2.16 to -2.08) | 1770(1210 to 2499) | 129.26(88.19 to 182.90) | 2748(1786 to 4027) | 67.53(43.89 to 98.96) | -2.04(-2.64 to -1.44) |
| **Philippines** | 3062(1850 to 4730) | 91.25(55.04 to 140.81) | 8004(5125 to 11740) | 81.09(51.87 to 118.67) | -0.39(-0.43 to -0.34) | 12611(8989 to 16820) | 388.92(278.47 to 517.25) | 28883(21438 to 37472) | 301.05(224.36 to 388.84) | -0.82(-0.84 to -0.80) | 2800(1865 to 4966) | 98.84(66.33 to 169.14) | 7713(6050 to 9732) | 86.55(67.59 to 108.63) | -0.38(-0.69 to -0.07) |
| **Poland** | 25042(16140 to 36382) | 431.57(277.52 to 629.10) | 17697(11065 to 26460) | 180.11(112.50 to 269.24) | -2.77(-2.86 to -2.68) | 75280(58091 to 95201) | 1334.07(1029.97 to 1683.44) | 34487(23919 to 47125) | 350.16(242.34 to 479.31) | -4.24(-4.30 to -4.17) | 14960(13072 to 17157) | 278.66(243.61 to 318.27) | 6490(5354 to 7826) | 64.95(53.53 to 78.49) | -4.56(-4.86 to -4.26) |
| **Portugal** | 7270(4535 to 10823) | 379.69(237.18 to 565.47) | 5457(3600 to 7719) | 179.16(116.98 to 255.41) | -2.41(-2.65 to -2.17) | 8981(6593 to 11889) | 479.01(351.76 to 633.89) | 8753(6427 to 11605) | 278.99(201.88 to 375.28) | -1.72(-1.76 to -1.69) | 2063(1710 to 2478) | 120.38(99.79 to 143.92) | 2557(2027 to 3150) | 69.46(54.59 to 86.77) | -1.54(-2.46 to -0.61) |
| **Puerto Rico** | 586(376 to 860) | 125.88(80.84 to 184.79) | 782(503 to 1165) | 87.33(55.76 to 131.36) | -1.18(-1.37 to -0.98) | 1845(1327 to 2466) | 394.74(283.26 to 528.63) | 1980(1362 to 2739) | 218.23(148.20 to 305.50) | -1.92(-2.00 to -1.84) | 914(771 to 1074) | 206.37(174.07 to 242.44) | 428(326 to 552) | 42.59(32.18 to 55.62) | -4.98(-5.99 to -3.96) |
| **Qatar** | 20(11 to 31) | 205.74(119.19 to 329.05) | 165(98 to 259) | 200.52(122.55 to 308.85) | -0.04(-0.33 to 0.26) | 33(19 to 51) | 360.77(219.03 to 549.00) | 274(169 to 421) | 355.12(229.06 to 525.46) | -0.08(-0.31 to 0.15) | 5(3 to 9) | 65.82(36.31 to 128.75) | 31(17 to 54) | 48.70(27.00 to 84.25) | -1.10(-1.67 to -0.53) |
| **Republic of Korea** | 7813(4844 to 11922) | 227.32(141.93 to 344.78) | 17292(10360 to 26388) | 136.73(82.23 to 208.16) | -1.51(-2.04 to -0.97) | 15434(11303 to 20258) | 474.37(351.63 to 617.33) | 24304(15154 to 37348) | 194.04(121.56 to 296.81) | -2.93(-3.15 to -2.71) | 1800(1266 to 2415) | 62.76(44.91 to 83.09) | 2716(1630 to 4297) | 22.49(13.61 to 35.30) | -3.33(-3.57 to -3.09) |
| **Republic of Moldova** | 2254(1423 to 3318) | 380.12(239.89 to 559.31) | 1190(762 to 1749) | 144.14(92.34 to 211.46) | -3.14(-3.39 to -2.88) | 3894(2951 to 5077) | 671.92(511.32 to 872.21) | 2626(1863 to 3602) | 320.62(228.52 to 438.01) | -2.36(-2.42 to -2.31) | 797(651 to 971) | 157.77(129.64 to 190.80) | 646(513 to 810) | 81.23(64.57 to 101.54) | -2.17(-2.75 to -1.58) |
| **Romania** | 14418(8973 to 21721) | 383.90(238.78 to 579.46) | 9767(6190 to 14770) | 196.37(124.56 to 296.68) | -2.15(-2.37 to -1.92) | 28627(22383 to 36056) | 782.30(611.80 to 984.85) | 18651(12781 to 26293) | 373.25(255.26 to 527.25) | -2.38(-2.44 to -2.32) | 4984(4069 to 6059) | 146.19(120.24 to 176.27) | 2763(2069 to 3628) | 54.12(40.25 to 71.52) | -3.17(-3.55 to -2.78) |
| **Russian Federation** | 50877(31655 to 76524) | 208.13(129.03 to 314.28) | 69956(43172 to 106723) | 207.38(128.03 to 315.63) | -0.01(-0.11 to 0.08) | 139102(101853 to 182337) | 591.80(436.12 to 773.06) | 143133(100911 to 195734) | 429.51(304.38 to 585.08) | -1.02(-1.12 to -0.93) | 25577(22057 to 29900) | 114.89(99.52 to 133.22) | 29482(25002 to 34696) | 91.04(77.49 to 106.69) | -0.76(-1.60 to 0.08) |
| **Rwanda** | 348(210 to 538) | 102.46(61.49 to 158.78) | 823(510 to 1223) | 107.42(66.48 to 159.48) | 0.10(-0.14 to 0.34) | 1894(1315 to 2597) | 556.70(384.96 to 762.75) | 3835(2774 to 5132) | 504.48(364.38 to 673.95) | -0.39(-0.54 to -0.23) | 562(339 to 1052) | 197.16(114.78 to 392.60) | 1072(574 to 2683) | 170.57(89.83 to 432.62) | -0.47(-0.61 to -0.32) |
| **Saint Kitts and Nevis** | 9(6 to 13) | 165.88(105.89 to 242.66) | 10(7 to 14) | 102.41(68.47 to 144.50) | -1.54(-1.60 to -1.49) | 54(43 to 67) | 1009.67(805.83 to 1248.49) | 35(26 to 45) | 369.09(276.06 to 475.97) | -3.25(-3.42 to -3.07) | 10(8 to 12) | 185.81(150.56 to 227.21) | 7(5 to 10) | 94.01(70.56 to 120.25) | -2.00(-3.25 to -0.73) |
| **Saint Lucia** | 14(9 to 21) | 120.40(74.11 to 178.28) | 27(18 to 40) | 89.09(58.70 to 130.43) | -0.95(-1.08 to -0.82) | 50(36 to 66) | 432.65(314.16 to 572.06) | 89(65 to 119) | 294.46(214.36 to 391.68) | -1.26(-1.33 to -1.19) | 11(9 to 14) | 105.28(81.67 to 132.24) | 20(15 to 26) | 67.74(51.12 to 87.73) | -1.43(-1.91 to -0.94) |
| **Saint Vincent and the Grenadines** | 12(8 to 18) | 127.99(81.92 to 188.12) | 23(16 to 32) | 121.69(82.79 to 170.10) | -0.17(-0.19 to -0.15) | 54(41 to 68) | 571.92(437.73 to 722.48) | 84(65 to 107) | 445.72(346.62 to 567.00) | -0.82(-0.88 to -0.76) | 14(11 to 17) | 154.83(126.78 to 187.03) | 24(20 to 30) | 138.55(113.01 to 167.92) | -0.35(-1.15 to 0.45) |
| **Samoa** | 10(6 to 15) | 93.99(58.87 to 141.52) | 15(10 to 21) | 83.69(55.06 to 121.41) | -0.36(-0.43 to -0.30) | 42(30 to 56) | 403.77(288.50 to 538.91) | 55(40 to 72) | 319.99(237.07 to 416.55) | -0.73(-0.78 to -0.67) | 5(3 to 8) | 53.38(33.24 to 86.28) | 7(4 to 11) | 42.92(26.30 to 69.66) | -0.69(-0.76 to -0.62) |
| **San Marino** | 14(8 to 21) | 296.15(173.40 to 465.56) | 13(8 to 20) | 152.84(92.03 to 234.42) | -2.11(-2.39 to -1.83) | 12(7 to 18) | 254.00(154.15 to 393.97) | 19(12 to 29) | 218.58(134.42 to 337.03) | -0.51(-0.65 to -0.38) | 2(1 to 4) | 49.89(31.03 to 75.55) | 3(2 to 5) | 27.08(14.75 to 44.55) | -2.07(-2.52 to -1.63) |
| **Sao Tome and Principe** | 8(5 to 12) | 95.34(59.20 to 141.90) | 12(7 to 17) | 92.65(58.88 to 134.94) | -0.13(-0.33 to 0.07) | 33(23 to 44) | 398.82(286.95 to 534.80) | 45(33 to 61) | 366.05(264.65 to 492.83) | -0.35(-0.53 to -0.16) | 9(5 to 21) | 116.61(63.20 to 284.74) | 12(7 to 22) | 106.49(62.31 to 208.96) | -0.31(-0.42 to -0.20) |
| **Saudi Arabia** | 1120(662 to 1754) | 172.52(102.17 to 269.85) | 2383(1406 to 3777) | 118.48(70.71 to 186.37) | -1.20(-1.32 to -1.07) | 1951(1206 to 2938) | 306.18(190.89 to 458.31) | 4814(2942 to 7352) | 249.00(154.90 to 375.63) | -0.68(-0.73 to -0.63) | 193(111 to 314) | 31.87(18.36 to 52.03) | 389(209 to 644) | 21.94(11.98 to 36.49) | -1.19(-1.28 to -1.11) |
| **Senegal** | 449(275 to 672) | 113.82(69.65 to 170.50) | 944(606 to 1375) | 101.19(64.75 to 147.05) | -0.46(-0.76 to -0.17) | 2143(1538 to 2875) | 561.00(404.08 to 750.16) | 4414(3261 to 5773) | 485.20(360.16 to 632.13) | -0.49(-0.77 to -0.20) | 664(373 to 1550) | 190.17(105.96 to 446.51) | 1249(703 to 2573) | 151.86(84.75 to 314.45) | -0.75(-0.83 to -0.67) |
| **Serbia** | 6417(3952 to 9834) | 412.37(253.45 to 628.13) | 5490(3733 to 7610) | 241.04(163.62 to 334.83) | -1.71(-1.90 to -1.53) | 9499(6763 to 12790) | 637.71(458.13 to 853.31) | 10169(7678 to 13336) | 447.64(336.61 to 589.54) | -1.14(-1.19 to -1.08) | 1992(1463 to 2654) | 163.78(119.44 to 219.29) | 2137(1556 to 2841) | 94.14(68.51 to 125.26) | -1.75(-2.09 to -1.42) |
| **Seychelles** | 7(4 to 11) | 98.79(61.33 to 148.95) | 11(7 to 16) | 76.46(49.96 to 109.45) | -0.85(-1.03 to -0.68) | 13(10 to 18) | 185.56(132.44 to 251.40) | 25(17 to 34) | 175.24(124.84 to 238.65) | -0.22(-0.27 to -0.17) | 2(1 to 3) | 27.32(17.97 to 40.43) | 3(2 to 4) | 21.06(12.91 to 32.70) | -0.77(-0.99 to -0.54) |
| **Sierra Leone** | 267(158 to 419) | 105.72(62.50 to 165.48) | 395(256 to 579) | 90.74(58.56 to 132.83) | -0.59(-0.75 to -0.43) | 1214(850 to 1651) | 489.61(342.99 to 664.75) | 1979(1496 to 2540) | 464.66(351.88 to 595.01) | -0.25(-0.34 to -0.16) | 327(183 to 755) | 141.91(78.58 to 331.65) | 569(304 to 1427) | 146.97(77.99 to 374.43) | 0.13(0.03 to 0.23) |
| **Singapore** | 486(293 to 750) | 185.28(111.90 to 285.39) | 1550(958 to 2345) | 136.55(84.79 to 205.50) | -0.82(-1.33 to -0.31) | 784(542 to 1076) | 308.78(215.89 to 419.94) | 2111(1348 to 3166) | 188.46(121.60 to 280.33) | -1.60(-1.65 to -1.55) | 137(106 to 174) | 56.97(44.50 to 71.91) | 271(186 to 395) | 25.07(17.39 to 35.97) | -2.63(-3.11 to -2.14) |
| **Slovakia** | 2741(1665 to 4232) | 342.17(208.21 to 528.70) | 2580(1677 to 3758) | 198.10(128.57 to 288.49) | -1.74(-1.84 to -1.64) | 4856(3574 to 6463) | 615.97(453.75 to 818.69) | 4885(3469 to 6631) | 377.51(267.98 to 512.83) | -1.58(-1.65 to -1.51) | 971(739 to 1251) | 126.88(96.53 to 163.26) | 792(562 to 1082) | 62.48(44.39 to 85.37) | -2.26(-2.41 to -2.12) |
| **Slovenia** | 1288(808 to 1918) | 408.90(256.06 to 611.22) | 1586(1044 to 2300) | 277.79(181.78 to 405.15) | -1.24(-1.38 to -1.10) | 2411(1807 to 3138) | 774.00(580.77 to 1005.47) | 2468(1773 to 3347) | 423.67(300.96 to 580.51) | -1.93(-2.03 to -1.82) | 576(481 to 685) | 184.84(153.78 to 220.11) | 411(313 to 526) | 65.57(49.33 to 84.94) | -3.30(-3.86 to -2.74) |
| **Solomon Islands** | 14(8 to 21) | 84.45(50.68 to 129.69) | 24(15 to 35) | 62.43(39.17 to 91.50) | -0.96(-1.11 to -0.80) | 65(45 to 87) | 415.49(291.24 to 561.41) | 114(84 to 150) | 308.72(229.10 to 404.69) | -0.95(-0.97 to -0.93) | 7(4 to 14) | 53.20(30.04 to 105.07) | 14(8 to 24) | 40.00(22.58 to 72.89) | -0.92(-1.03 to -0.80) |
| **Somalia** | 230(139 to 354) | 98.04(58.86 to 151.82) | 526(326 to 789) | 76.52(47.29 to 114.73) | -0.86(-1.06 to -0.66) | 1130(803 to 1543) | 482.60(341.61 to 659.70) | 3217(2348 to 4238) | 466.03(340.22 to 612.37) | -0.16(-0.23 to -0.09) | 381(192 to 986) | 194.43(94.69 to 528.62) | 1012(480 to 2405) | 179.85(82.43 to 455.75) | -0.23(-0.32 to -0.14) |
| **South Africa** | 1553(942 to 2374) | 62.86(38.16 to 95.82) | 3762(2407 to 5496) | 66.09(42.40 to 96.14) | 0.15(-0.03 to 0.32) | 8060(5887 to 10635) | 328.90(240.95 to 432.81) | 18017(13718 to 22980) | 319.91(244.74 to 406.30) | -0.09(-0.12 to -0.06) | 1933(1373 to 2891) | 84.81(60.11 to 126.97) | 5153(4167 to 6193) | 99.60(80.69 to 119.43) | 0.50(0.13 to 0.86) |
| **South Sudan** | 370(218 to 578) | 116.47(68.88 to 181.15) | 406(255 to 609) | 99.09(61.86 to 148.12) | -0.70(-0.98 to -0.42) | 1344(907 to 1861) | 424.75(287.06 to 587.95) | 2005(1432 to 2682) | 493.76(353.57 to 660.25) | 0.41(0.34 to 0.48) | 502(268 to 1128) | 176.58(93.47 to 397.60) | 643(344 to 1553) | 181.10(95.28 to 443.38) | 0.08(-0.02 to 0.17) |
| **Spain** | 32284(20297 to 48122) | 436.00(274.31 to 650.34) | 19800(13509 to 27954) | 164.68(111.15 to 234.31) | -3.09(-3.30 to -2.87) | 38316(28565 to 50304) | 520.70(387.86 to 683.86) | 29174(21780 to 38198) | 230.61(168.85 to 306.82) | -2.58(-2.65 to -2.52) | 9609(7997 to 11434) | 135.54(112.58 to 161.22) | 9532(7415 to 11642) | 62.99(48.92 to 77.80) | -2.38(-2.64 to -2.12) |
| **Sri Lanka** | 1993(1231 to 2985) | 159.36(98.51 to 238.15) | 3486(2311 to 5020) | 97.18(64.55 to 139.41) | -1.59(-1.70 to -1.48) | 4482(3184 to 6120) | 381.75(273.10 to 518.11) | 7105(5163 to 9528) | 204.61(149.42 to 273.00) | -2.01(-2.07 to -1.95) | 1511(978 to 2376) | 144.85(94.55 to 225.07) | 1817(1141 to 2748) | 56.30(35.59 to 84.75) | -3.03(-3.22 to -2.85) |
| **Sudan** | 1633(939 to 2624) | 141.68(81.53 to 227.03) | 2382(1420 to 3653) | 107.36(64.21 to 164.26) | -0.84(-1.10 to -0.57) | 4117(2545 to 6253) | 360.71(223.22 to 546.72) | 7067(4537 to 10356) | 322.67(208.45 to 470.50) | -0.40(-0.54 to -0.26) | 391(218 to 650) | 35.89(19.84 to 61.05) | 647(369 to 1074) | 30.82(17.54 to 51.71) | -0.51(-0.58 to -0.44) |
| **Suriname** | 53(33 to 80) | 168.62(105.20 to 255.02) | 90(59 to 130) | 113.08(74.69 to 163.62) | -1.28(-1.36 to -1.20) | 245(181 to 319) | 799.08(589.67 to 1042.18) | 380(294 to 483) | 480.40(371.36 to 610.40) | -1.64(-1.71 to -1.56) | 55(38 to 77) | 187.06(129.06 to 265.42) | 91(59 to 136) | 119.42(77.48 to 178.85) | -1.36(-1.68 to -1.04) |
| **Sweden** | 4900(3027 to 7619) | 247.10(151.88 to 384.55) | 4055(2510 to 6089) | 147.78(90.46 to 224.95) | -1.66(-1.70 to -1.61) | 9123(6474 to 12413) | 448.40(312.52 to 619.62) | 6958(4707 to 9796) | 245.10(160.69 to 353.34) | -1.95(-2.00 to -1.89) | 1870(1533 to 2285) | 88.03(71.48 to 108.91) | 1483(1152 to 1869) | 46.96(35.98 to 60.52) | -2.12(-3.14 to -1.09) |
| **Switzerland** | 4496(2730 to 6943) | 346.37(209.37 to 537.18) | 4295(2698 to 6441) | 197.16(122.12 to 298.59) | -1.78(-2.01 to -1.55) | 4440(2975 to 6339) | 337.74(222.34 to 488.54) | 6265(4251 to 9076) | 281.51(186.62 to 415.72) | -0.65(-0.84 to -0.46) | 1000(797 to 1247) | 71.87(56.74 to 90.49) | 1350(999 to 1737) | 51.60(37.66 to 68.11) | -1.10(-1.72 to -0.47) |
| **Syrian Arab Republic** | 1054(618 to 1691) | 165.45(97.05 to 265.43) | 1981(1168 to 3139) | 114.97(68.26 to 181.16) | -1.18(-1.45 to -0.92) | 1941(1189 to 2951) | 309.99(191.12 to 469.17) | 4750(2953 to 7142) | 282.39(177.60 to 420.71) | -0.33(-0.43 to -0.24) | 187(107 to 320) | 31.77(18.27 to 55.07) | 421(229 to 700) | 27.07(14.84 to 46.75) | -0.53(-0.67 to -0.40) |
| **Taiwan (Province of China)** | 2778(1854 to 3999) | 134.15(90.02 to 191.94) | 4688(2961 to 7034) | 82.67(52.39 to 123.42) | -1.56(-1.71 to -1.41) | 8642(6389 to 11472) | 437.26(326.96 to 574.08) | 9292(6308 to 13240) | 164.73(112.30 to 233.83) | -3.13(-3.24 to -3.01) | 937(673 to 1289) | 53.27(39.57 to 71.02) | 1227(855 to 1720) | 21.71(15.20 to 30.33) | -2.89(-3.52 to -2.26) |
| **Tajikistan** | 369(210 to 599) | 106.64(60.75 to 173.25) | 551(320 to 873) | 73.32(42.85 to 115.36) | -1.24(-1.37 to -1.12) | 956(561 to 1485) | 281.45(165.81 to 436.61) | 2239(1376 to 3312) | 304.83(190.37 to 445.69) | 0.14(0.02 to 0.26) | 129(79 to 218) | 39.74(24.26 to 67.99) | 228(136 to 356) | 34.15(20.59 to 53.02) | -0.52(-0.73 to -0.31) |
| **Thailand** | 5609(3470 to 8499) | 134.86(83.23 to 204.99) | 14128(9368 to 19974) | 99.68(66.16 to 140.75) | -0.96(-1.06 to -0.86) | 11842(8266 to 16191) | 292.34(204.70 to 399.56) | 28855(20805 to 38602) | 204.88(148.41 to 273.10) | -1.15(-1.19 to -1.12) | 3425(2095 to 5429) | 90.32(55.01 to 143.48) | 8106(5173 to 12410) | 58.43(37.28 to 89.37) | -1.40(-1.64 to -1.17) |
| **Timor-Leste** | 34(20 to 54) | 124.25(72.57 to 193.14) | 120(74 to 177) | 108.13(67.23 to 159.85) | -0.53(-0.78 to -0.29) | 93(60 to 136) | 354.96(230.76 to 513.97) | 376(261 to 517) | 350.30(244.36 to 481.31) | -0.12(-0.22 to -0.02) | 25(12 to 75) | 104.84(48.68 to 306.15) | 108(54 to 263) | 110.65(54.98 to 266.92) | 0.18(0.02 to 0.34) |
| **Togo** | 147(89 to 223) | 104.74(63.19 to 158.75) | 400(256 to 591) | 90.95(58.06 to 134.25) | -0.60(-0.80 to -0.40) | 683(485 to 915) | 502.48(358.77 to 670.15) | 1927(1449 to 2491) | 452.90(341.74 to 583.38) | -0.34(-0.48 to -0.21) | 219(115 to 547) | 180.70(93.88 to 462.60) | 597(348 to 1264) | 165.47(93.95 to 367.31) | -0.28(-0.40 to -0.17) |
| **Tokelau** | 0(0 to 0) | 86.72(53.62 to 132.71) | 0(0 to 0) | 69.61(45.13 to 102.14) | -0.71(-0.78 to -0.64) | 1(1 to 1) | 389.79(277.94 to 525.24) | 0(0 to 1) | 254.31(183.85 to 339.56) | -1.38(-1.46 to -1.30) | 0(0 to 0) | 67.71(40.79 to 115.75) | 0(0 to 0) | 38.92(23.58 to 62.55) | -1.78(-1.88 to -1.68) |
| **Tonga** | 8(5 to 12) | 113.47(70.52 to 171.06) | 10(6 to 14) | 100.02(66.33 to 142.35) | -0.39(-0.43 to -0.34) | 35(25 to 48) | 525.29(375.38 to 705.95) | 37(28 to 48) | 383.28(289.63 to 495.30) | -1.00(-1.08 to -0.92) | 5(3 to 10) | 81.34(48.08 to 157.01) | 6(3 to 9) | 58.58(36.08 to 97.85) | -1.07(-1.32 to -0.82) |
| **Trinidad and Tobago** | 183(117 to 267) | 172.84(111.03 to 251.20) | 339(226 to 480) | 128.80(86.28 to 182.00) | -0.94(-1.04 to -0.84) | 811(623 to 1021) | 765.01(588.51 to 962.16) | 1144(864 to 1457) | 437.47(331.30 to 556.28) | -1.80(-1.86 to -1.73) | 156(126 to 190) | 157.27(127.98 to 190.77) | 200(149 to 262) | 78.97(58.87 to 103.38) | -2.31(-2.88 to -1.75) |
| **Tunisia** | 985(568 to 1565) | 150.05(86.52 to 237.90) | 2144(1270 to 3314) | 120.31(71.48 to 185.39) | -0.68(-0.73 to -0.62) | 1996(1206 to 3039) | 307.07(186.88 to 465.52) | 4798(2995 to 7240) | 272.60(171.18 to 409.25) | -0.42(-0.51 to -0.34) | 177(96 to 313) | 28.99(15.77 to 52.63) | 394(218 to 684) | 23.17(12.83 to 40.25) | -0.77(-0.91 to -0.63) |
| **Turkey** | 7022(4197 to 11006) | 166.52(99.43 to 261.19) | 17883(11201 to 26906) | 145.96(91.53 to 219.18) | -0.42(-0.60 to -0.24) | 15095(9580 to 22259) | 368.91(234.64 to 542.61) | 40206(27022 to 56897) | 332.10(223.88 to 468.60) | -0.33(-0.40 to -0.26) | 3381(2198 to 5056) | 88.93(57.38 to 133.80) | 5777(3824 to 8437) | 50.67(33.55 to 73.91) | -1.78(-2.10 to -1.46) |
| **Turkmenistan** | 293(187 to 429) | 119.65(75.44 to 176.67) | 454(295 to 675) | 88.25(57.16 to 131.05) | -0.96(-1.03 to -0.89) | 1178(898 to 1522) | 503.39(384.58 to 648.15) | 1710(1283 to 2236) | 345.16(260.34 to 447.97) | -1.22(-1.24 to -1.20) | 215(170 to 271) | 97.37(77.27 to 122.50) | 294(225 to 380) | 63.17(48.42 to 81.51) | -1.38(-1.63 to -1.12) |
| **Tuvalu** | 1(0 to 1) | 83.80(52.37 to 125.04) | 1(1 to 2) | 74.77(48.15 to 108.90) | -0.35(-0.39 to -0.32) | 4(3 to 5) | 449.53(328.73 to 588.61) | 4(3 to 6) | 325.35(242.79 to 424.58) | -1.03(-1.09 to -0.96) | 1(0 to 1) | 70.07(43.56 to 113.00) | 1(0 to 1) | 49.02(29.76 to 77.44) | -1.15(-1.22 to -1.07) |
| **Uganda** | 714(437 to 1076) | 94.14(57.43 to 141.97) | 1591(1023 to 2387) | 96.23(61.53 to 144.57) | -0.05(-0.30 to 0.19) | 3732(2679 to 4959) | 492.07(353.03 to 653.88) | 7331(5403 to 9619) | 444.26(327.50 to 582.76) | -0.34(-0.42 to -0.25) | 843(481 to 1578) | 125.81(70.02 to 243.00) | 1510(907 to 2483) | 104.28(61.05 to 177.17) | -0.61(-0.66 to -0.56) |
| **Ukraine** | 12763(7251 to 20097) | 127.57(72.36 to 201.25) | 9943(5786 to 15792) | 92.70(53.85 to 147.26) | -1.05(-1.21 to -0.89) | 27836(17488 to 40794) | 283.85(179.82 to 414.00) | 24861(16151 to 35808) | 232.96(151.65 to 335.12) | -0.64(-0.69 to -0.58) | 4017(2994 to 5341) | 42.08(31.56 to 55.55) | 4016(2897 to 5415) | 37.84(27.32 to 50.96) | -0.36(-0.80 to 0.07) |
| **United Arab Emirates** | 60(35 to 95) | 162.91(95.89 to 258.58) | 502(294 to 791) | 125.96(75.27 to 194.73) | -0.84(-1.04 to -0.65) | 87(52 to 134) | 241.80(146.50 to 371.84) | 1026(607 to 1593) | 278.56(171.84 to 419.48) | 0.45(0.18 to 0.72) | 39(24 to 62) | 119.98(72.18 to 190.84) | 152(90 to 248) | 72.83(42.35 to 121.08) | -1.56(-3.71 to 0.64) |
| **United Kingdom** | 69987(43155 to 105507) | 583.08(359.09 to 881.19) | 84738(51950 to 126822) | 518.75(317.08 to 779.24) | -0.41(-0.90 to 0.08) | 70468(52633 to 91890) | 576.93(427.51 to 756.75) | 51800(38649 to 67475) | 305.46(225.13 to 402.07) | -2.03(-2.08 to -1.98) | 17770(15602 to 20126) | 144.12(125.68 to 163.95) | 18499(15815 to 20783) | 99.12(84.78 to 112.10) | -1.02(-1.51 to -0.53) |
| **United Republic of Tanzania** | 1194(727 to 1882) | 90.86(55.24 to 142.84) | 2820(1811 to 4160) | 97.56(62.44 to 143.77) | 0.12(-0.06 to 0.30) | 5558(3903 to 7625) | 422.75(296.70 to 579.16) | 12558(9278 to 16465) | 435.37(320.88 to 571.10) | 0.09(-0.08 to 0.25) | 1605(906 to 3443) | 139.95(77.92 to 303.78) | 3404(1921 to 7198) | 131.26(73.29 to 282.25) | -0.19(-0.30 to -0.09) |
| **United States of America** | 54188(33389 to 82287) | 126.66(77.87 to 192.91) | 72346(45169 to 109198) | 91.41(56.94 to 138.08) | -1.05(-1.14 to -0.96) | 44(31 to 58) | 234.15(161.85 to 323.06) | 114451(77322 to 162557) | 144.39(97.28 to 205.42) | -1.52(-1.63 to -1.40) | 33869(29662 to 37951) | 78.83(68.89 to 88.50) | 36977(31149 to 42458) | 45.78(38.68 to 52.57) | -1.67(-1.95 to -1.39) |
| **United States Virgin Islands** | 12(8 to 18) | 117.74(74.39 to 178.32) | 26(17 to 37) | 101.34(67.08 to 145.26) | -0.51(-0.55 to -0.47) | 100485(69898 to 137897) | 427.90(307.85 to 572.03) | 79(58 to 105) | 311.49(226.96 to 413.68) | -1.03(-1.07 to -0.99) | 7(5 to 11) | 79.12(52.63 to 116.32) | 9(6 to 13) | 35.94(23.08 to 52.87) | -2.54(-2.74 to -2.33) |
| **Uruguay** | 777(522 to 1095) | 149.44(100.50 to 210.68) | 972(653 to 1382) | 139.80(93.84 to 199.61) | -0.18(-0.39 to 0.03) | 2738(2075 to 3509) | 527.41(399.31 to 676.44) | 2725(2052 to 3511) | 385.06(287.50 to 499.57) | -1.01(-1.06 to -0.96) | 639(526 to 762) | 124.97(102.76 to 148.95) | 823(678 to 983) | 106.56(87.73 to 127.91) | -0.49(-0.80 to -0.17) |
| **Uzbekistan** | 1207(696 to 1920) | 83.83(48.08 to 134.14) | 2483(1514 to 3780) | 74.78(45.85 to 113.09) | -0.37(-0.48 to -0.26) | 3630(2332 to 5224) | 258.94(167.16 to 371.20) | 8399(5684 to 11775) | 259.55(177.47 to 361.21) | -0.05(-0.28 to 0.18) | 514(362 to 714) | 37.91(26.92 to 52.27) | 937(640 to 1326) | 30.78(21.45 to 43.01) | -0.72(-0.98 to -0.45) |
| **Vanuatu** | 8(5 to 12) | 108.71(65.33 to 165.38) | 17(11 to 25) | 82.52(52.39 to 121.84) | -0.86(-0.95 to -0.78) | 41(28 to 56) | 577.03(403.71 to 783.73) | 81(60 to 105) | 412.35(309.62 to 533.74) | -1.06(-1.14 to -0.97) | 6(3 to 13) | 93.83(49.19 to 234.92) | 12(7 to 27) | 71.54(38.62 to 165.67) | -0.87(-0.89 to -0.85) |
| **Venezuela (Bolivarian Republic of)** | 3464(2125 to 5394) | 301.79(184.71 to 470.95) | 5472(3603 to 7929) | 141.72(93.21 to 204.96) | -2.42(-2.51 to -2.34) | 8260(6016 to 10989) | 731.41(533.00 to 971.91) | 18379(13776 to 23818) | 479.85(359.78 to 621.37) | -1.38(-1.45 to -1.30) | 2875(2395 to 3451) | 269.91(224.53 to 323.89) | 5472(4103 to 7054) | 150.14(112.84 to 193.04) | -1.83(-2.32 to -1.34) |
| **Viet Nam** | 4781(2835 to 7462) | 93.78(55.70 to 146.19) | 8928(5796 to 12992) | 71.09(46.29 to 102.88) | -0.92(-1.07 to -0.77) | 11823(8048 to 16612) | 237.93(162.61 to 333.11) | 20534(14353 to 27934) | 166.47(117.75 to 224.62) | -1.20(-1.29 to -1.10) | 2486(1385 to 4596) | 52.61(29.29 to 97.03) | 3876(2300 to 6018) | 34.67(20.52 to 53.99) | -1.35(-1.41 to -1.30) |
| **Yemen** | 909(531 to 1430) | 147.83(86.26 to 233.68) | 1738(1061 to 2707) | 103.90(63.56 to 161.17) | -1.14(-1.30 to -0.98) | 2271(1428 to 3337) | 378.97(238.49 to 555.89) | 6306(4213 to 8926) | 381.74(256.60 to 537.69) | -0.03(-0.13 to 0.07) | 207(117 to 341) | 36.82(20.53 to 62.18) | 577(344 to 921) | 37.08(22.14 to 60.33) | -0.01(-0.11 to 0.10) |
| **Zambia** | 327(194 to 507) | 103.15(60.89 to 159.84) | 700(448 to 1049) | 93.66(59.94 to 139.67) | -0.46(-0.73 to -0.19) | 1614(1140 to 2174) | 509.60(358.72 to 686.22) | 3218(2404 to 4184) | 431.45(321.80 to 561.29) | -0.59(-0.70 to -0.47) | 453(276 to 899) | 162.29(97.24 to 326.27) | 826(511 to 1293) | 127.33(77.11 to 205.72) | -0.76(-0.94 to -0.59) |
| **Zimbabwe** | 668(426 to 982) | 133.76(85.18 to 196.34) | 848(546 to 1230) | 105.48(67.76 to 152.63) | -0.82(-0.98 to -0.67) | 3481(2616 to 4542) | 715.54(539.35 to 930.17) | 4959(3849 to 6278) | 637.23(495.38 to 802.92) | -0.42(-0.48 to -0.37) | 768(497 to 1138) | 176.14(113.70 to 261.85) | 1178(720 to 1820) | 169.57(103.16 to 263.04) | -0.07(-0.29 to 0.15) |

Rates are reported per 100,000 person-years. Data in parentheses are 95% uncertainty intervals for cases and age-standardized rates of incidence, prevalence and DALYs, and 95% confidence intervals for AAPCs.

Abbreviations: DALYs, Disability-Adjusted Life Years; AAPC, average annual percent change; UI, uncertainty interval.

**S2 Table.** **Forecast of Inguinal, femoral, and abdominal hernias among Older Adults age-standardised Incidence, Prevalence and DALYs rates and total cases globally, to 2035, with 95% uncertainty intervals.**

|  | **Incident cases** | **ASR of Incidence** | **Prevalent cases** | **ASR of Prevalence** | **DALYs cases** | **ASR of DALYs** |
| --- | --- | --- | --- | --- | --- | --- |
| **Both** | | | | | | |
| **2022** | 1452885(1287316 to 1618455) | 129.83(120.69 to 138.98) | 3676398(3490327 to 3862469) | 330(321 to 340) | 803497(760357 to 846637) | 74.49(72.14 to 76.84) |
| **2023** | 1517615(1308208 to 1727021) | 131.29(117.91 to 144.66) | 3803546(3577033 to 4030060) | 331(318 to 344) | 815621(760930 to 870313) | 73.24(69.63 to 76.85) |
| **2024** | 1591052(1315424 to 1866680) | 132.85(113.87 to 151.84) | 3946962(3661124 to 4232801) | 332(314 to 350) | 830940(758989 to 902891) | 72.06(66.88 to 77.24) |
| **2025** | 1666723(1303531 to 2029915) | 134.53(108.84 to 160.22) | 4090020(3727136 to 4452904) | 333(309 to 357) | 846149(752176 to 940123) | 70.92(63.96 to 77.88) |
| **2026** | 1745499(1273314 to 2217685) | 136.29(102.92 to 169.65) | 4233956(3776592 to 4691320) | 334(303 to 365) | 861510(741269 to 981752) | 69.83(60.93 to 78.73) |
| **2027** | 1827700(1224486 to 2430914) | 138.12(96.16 to 180.08) | 4377924(3809408 to 4946441) | 336(297 to 374) | 877204(726761 to 1027648) | 68.78(57.80 to 79.76) |
| **2028** | 1918401(1158975 to 2677826) | 140.04(88.60 to 191.48) | 4532013(3835078 to 5228949) | 337(291 to 384) | 894481(709893 to 1079069) | 67.77(54.60 to 80.93) |
| **2029** | 2018589(1074053 to 2963124) | 142.05(80.25 to 203.86) | 4696336(3852402 to 5540270) | 339(284 to 395) | 912945(690209 to 1135682) | 66.80(51.35 to 82.26) |
| **2030** | 2126811(964872 to 3288750) | 144.15(71.09 to 217.22) | 4865507(3855598 to 5875416) | 341(276 to 406) | 932035(667112 to 1196958) | 65.89(48.06 to 83.72) |
| **2031** | 2243393(826979 to 3659807) | 146.33(61.11 to 231.54) | 5039111(3843357 to 6234864) | 343(268 to 418) | 951756(640435 to 1263078) | 65.01(44.73 to 85.29) |
| **2032** | 2366993(654500 to 4079487) | 148.57(50.29 to 246.84) | 5212055(3811044 to 6613066) | 345(259 to 431) | 971873(609829 to 1333916) | 64.16(41.36 to 86.97) |
| **2033** | 2499769(441996 to 4557541) | 150.88(38.61 to 263.15) | 5385588(3759963 to 7011213) | 347(249 to 445) | 992727(575415 to 1410040) | 63.35(37.96 to 88.75) |
| **2034** | 2644259(181564 to 5106955) | 153.27(26.05 to 280.48) | 5561610(3690613 to 7432607) | 350(239 to 460) | 1013945(536685 to 1491205) | 62.59(34.54 to 90.63) |
| **2035** | 2800887(0 to 5739342) | 155.73(12.59 to 298.87) | 5737595(3599989 to 7875201) | 352(229 to 475) | 1035231(493172 to 1577290) | 61.86(31.10 to 92.62) |
| **Male** | | | | | | |
| **2022** | 1241186(1097153 to 1385219) | 241.95(224.61 to 259.28) | 2800340(2661669 to 2939012) | 554(538 to 570) | 500211(473478 to 526943) | 105.59(102.13 to 109.05) |
| **2023** | 1299218(1116179 to 1482256) | 244.90(219.44 to 270.35) | 2897659(2727234 to 3068084) | 555(532 to 577) | 509362(473894 to 544829) | 103.98(98.41 to 109.55) |
| **2024** | 1365541(1123557 to 1607525) | 248.07(211.86 to 284.29) | 3008497(2791254 to 3225740) | 555(525 to 586) | 520894(472445 to 569344) | 102.46(94.28 to 110.64) |
| **2025** | 1434036(1114025 to 1754047) | 251.46(202.37 to 300.55) | 3118840(2840821 to 3396860) | 557(516 to 598) | 532379(467465 to 597293) | 101.03(89.89 to 112.16) |
| **2026** | 1505536(1088159 to 1922913) | 255.03(191.20 to 318.87) | 3229804(2877373 to 3582235) | 558(506 to 611) | 543993(459483 to 628504) | 99.65(85.28 to 114.02) |
| **2027** | 1580378(1045562 to 2115195) | 258.76(178.39 to 339.12) | 3340990(2901096 to 3780884) | 560(495 to 626) | 555877(448800 to 662953) | 98.33(80.49 to 116.17) |
| **2028** | 1663231(987791 to 2338670) | 262.67(164.03 to 361.30) | 3460613(2919606 to 4001619) | 562(483 to 642) | 569062(436323 to 701802) | 97.08(75.57 to 118.58) |
| **2029** | 1754971(912112 to 2597829) | 266.76(148.13 to 385.40) | 3588299(2931532 to 4245065) | 565(470 to 659) | 583311(421677 to 744946) | 95.91(70.55 to 121.26) |
| **2030** | 1854327(813895 to 2894758) | 271.03(130.64 to 411.43) | 3719585(2932155 to 4507015) | 567(457 to 678) | 598135(404301 to 791970) | 94.80(65.44 to 124.16) |
| **2031** | 1961674(688804 to 3234543) | 275.46(111.53 to 439.39) | 3854174(2920482 to 4787867) | 570(442 to 698) | 613529(383987 to 843071) | 93.75(60.24 to 127.27) |
| **2032** | 2075901(531146 to 3620656) | 280.03(90.75 to 469.30) | 3988122(2892933 to 5083311) | 573(427 to 719) | 629307(360392 to 898222) | 92.74(54.93 to 130.55) |
| **2033** | 2199108(335428 to 4062789) | 284.74(68.26 to 501.22) | 4122622(2850604 to 5394640) | 576(410 to 742) | 645781(333512 to 958051) | 91.78(49.55 to 134.02) |
| **2034** | 2333622(99774 to 4573562) | 289.61(44.02 to 535.21) | 4258946(2793701 to 5724192) | 579(393 to 766) | 662768(302915 to 1022622) | 90.89(44.10 to 137.68) |
| **2035** | 2479911(0 to 5164666) | 294.64(17.98 to 571.30) | 4394998(2719825 to 6070172) | 583(374 to 791) | 680038(268119 to 1091957) | 90.05(38.59 to 141.52) |
| **Female** | | | | | | |
| **2022** | 197451(177690 to 217212) | 32.58(30.59 to 34.57) | 831109(771274 to 890945) | 137(132 to 143) | 296319(273587 to 319052) | 48.74(46.72 to 50.75) |
| **2023** | 206781(180965 to 232597) | 33.07(29.96 to 36.17) | 860561(790827 to 930295) | 138(131 to 145) | 300383(273662 to 327103) | 47.91(45.07 to 50.76) |
| **2024** | 217387(182361 to 252414) | 33.59(29.03 to 38.15) | 893699(809752 to 977645) | 138(129 to 148) | 305469(272731 to 338207) | 47.11(43.23 to 50.99) |
| **2025** | 228544(181299 to 275789) | 34.15(27.85 to 40.45) | 927129(824734 to 1029524) | 139(127 to 151) | 310597(270026 to 351167) | 46.34(41.27 to 51.40) |
| **2026** | 240369(177861 to 302877) | 34.74(26.44 to 43.04) | 961043(835867 to 1086219) | 140(125 to 155) | 315884(265812 to 365955) | 45.59(39.23 to 51.95) |
| **2027** | 252966(171987 to 333945) | 35.36(24.81 to 45.91) | 995185(843026 to 1147343) | 141(123 to 159) | 321391(260277 to 382506) | 44.87(37.13 to 52.61) |
| **2028** | 266915(163801 to 370029) | 36.01(22.95 to 49.06) | 1031523(848018 to 1215028) | 142(120 to 163) | 327365(253707 to 401023) | 44.16(34.97 to 53.36) |
| **2029** | 282326(152862 to 411790) | 36.69(20.88 to 52.50) | 1070243(850646 to 1289840) | 143(117 to 168) | 333639(245953 to 421326) | 43.48(32.78 to 54.18) |
| **2030** | 299113(138501 to 459726) | 37.41(18.59 to 56.24) | 1110402(849790 to 1371015) | 144(113 to 174) | 340154(236932 to 443377) | 42.83(30.57 to 55.08) |
| **2031** | 317336(120018 to 514653) | 38.16(16.05 to 60.27) | 1151941(845114 to 1458768) | 145(110 to 180) | 346983(226639 to 467327) | 42.20(28.33 to 56.06) |
| **2032** | 336961(96538 to 577384) | 38.94(13.27 to 64.61) | 1193954(835732 to 1552177) | 146(106 to 186) | 354106(214989 to 493223) | 41.59(26.08 to 57.10) |
| **2033** | 358353(67170 to 649537) | 39.75(10.23 to 69.27) | 1236658(821839 to 1651476) | 147(102 to 192) | 361565(201952 to 521177) | 41.00(23.80 to 58.20) |
| **2034** | 381766(30617 to 732915) | 40.59(6.93 to 74.26) | 1280297(803292 to 1757302) | 148(98 to 199) | 369144(187291 to 550997) | 40.44(21.52 to 59.35) |
| **2035** | 407350(0 to 829501) | 41.47(3.34 to 79.60) | 1324387(779354 to 1869419) | 150(93 to 207) | 376806(170865 to 582748) | 39.89(19.23 to 60.55) |

Abbreviations: DALYs, Disability-Adjusted Life Years; AAPC, average annual percent change; UI, uncertainty interval; CI, confidence interval.
