# Supplementary material for: Global burden and future trends of inguinal, femoral, and abdominal hernia in older adults: A systematic analysis from the Global Burden of Disease Study 2021
Source: PLoS One. 2025 Jun 6;20(6):e0323790. doi: 10.1371/journal.pone.0323790 (PMC12143580; doi:10.1371/journal.pone.0323790)
Supplement: Attachment — Submitted filename: Response to Reviewers.docx [file pone.0323790.s004.docx]

**11 February 2025**

Lovenish Bains, MS, FNB, FACS, FRCS (Glas), FICS, FIAGES, PhD

Academic Editor

PLOS ONE

Dear Editor and Reviewers:

We really appreciate the critical reading of our manuscript entitled “Global burden and future trends of inguinal, femoral, and abdominal hernia in older adults: a systematic analysis from the Global Burden of Disease Study 2021” (Manuscript Number: PONE-D-24-46904). We thank you for all the valuable suggestions received from the reviewers and editor. We have carefully considered the comments and have revised the manuscript accordingly. The responses to the comments are listed one by one as follows. (Please see below, the responses to the editor and reviewers are represented in red font, while the modifications made to the original text are shown in blue font.) We are looking forward to hearing about your decision.

**Journal Requirements:**

1. Please ensure that your manuscript meets PLOS ONE's style requirements, including those for file naming.

**Answer:** Thank you for your comments. I have made the necessary formatting revisions as per your requirements and have resubmitted the manuscript.

The author’s institution has released new formatting guidelines; therefore, I have adjusted the author affiliation format accordingly while ensuring that these changes do not affect the content of the manuscript. Affiliations 1 and 2 represent the same institution.

*Line 10-12*

*“1 The Gastrointestinal, Pancreatic, Hernia and Abdominal Wall Surgery Department of Shanxi Provincial People's Hospital, Shanxi Medical University, Taiyuan, China;*

*2 The Fifth Clinical Medical College, Shanxi Medical University, Taiyuan, China;”*

2. When completing the data availability statement of the submission form, you indicated that you will make your data available on acceptance. We strongly recommend all authors decide on a data sharing plan before acceptance, as the process can be lengthy and hold up publication timelines. Please note that, though access restrictions are acceptable now, your entire data will need to be made freely accessible if your manuscript is accepted for publication. This policy applies to all data except where public deposition would breach compliance with the protocol approved by your research ethics board. If you are unable to adhere to our open data policy, please kindly revise your statement to explain your reasoning and we will seek the editor's input on an exemption. Please be assured that, once you have provided your new statement, the assessment of your exemption will not hold up the peer review process.

Answer: All relevant data are within the manuscript and its Supporting Information files.

3. PLOS requires an ORCID iD for the corresponding author in Editorial Manager on papers submitted after December 6th, 2016. Please ensure that you have an ORCID iD and that it is validated in Editorial Manager. To do this, go to ‘Update my Information’ (in the upper left-hand corner of the main menu),

**Answer:** Thank you for your guidance. I have now uploaded the ORCID iD for the corresponding author and completed the necessary information update.

**Reviewers' comments:**

**Reviewer #1:**

Originality 5

Brevity and focus 5

Evaluation of analyses 5

Interpretation of results 5

Writing style 5

References 3

Accuracy 3

Language 4
(over 5)

1. please define aim

**Answer:** Thank you for highlighting the need to better define the objectives of our study. In response, we have added an "Objective" section to the abstract to more clearly articulate the aim of our research. Additionally, we have made corresponding updates in the introduction section to further refine and clarify the study objectives. We appreciate your insightful feedback, which has helped improve the focus and clarity of our work.

*Line 24-27*

*“Objective: This study aims to comprehensively evaluate the global, regional, and national burden, trends, and health inequalities of inguinal, femoral, and abdominal hernias among older adults from 1990 to 2021, conduct predictive analyses, and provide insights to inform future public health strategies.*

*Line 73-89*

*“In this background, our study aims to comprehensively evaluate the global, regional, and national burden of inguinal, femoral, and abdominal hernias among older adults from 1990 to 2021, utilizing data from the GBD 2021.*

*The analysis focuses on trends in incidence, prevalence, and DALYs across 204 countries and territories, stratified by geographic regions, SDI levels, age groups, and gender. By identifying and quantifying disparities in the disease burden, particularly across different SDI regions, our study seeks to provide actionable insights into the unequal distribution of healthcare resources and outcomes. The findings aim to inform the development of tailored public health policies and strategies that prioritize the needs of older adults, especially in low- and lower-middle SDI regions where the burden is disproportionately high. Additionally, the results are intended to serve as a baseline for policymakers, healthcare providers, and global health organizations to design evidence-based interventions, allocate resources effectively, and address health disparities in aging populations. The ultimate goal is to implement a series of effective measures to alleviate the burden of hernias among older adults, thereby reducing the strain on societal healthcare systems.”*

2. please add more update studies for the references

**Answer:** We appreciate your suggestion to include more updated studies for the references. As you mentioned, incorporating updated references into the study adds greater significance to the research. Therefore, I have updated some of the references accordingly.

*Line 569-717*

[2] *Hamilton J, Kushner B, Holden S, Holden T. Age-Related Risk Factors in Ventral Hernia Repairs: A Review and Call to Action. J Surg Res. 2021;266:180-191. doi:10.1016/j.jss.2021.04.004*
[3] *Ceresoli M, Carissimi F, Nigro A, et al. Emergency hernia repair in the elderly: multivariate analysis of morbidity and mortality from an Italian registry. Hernia. 2022;26(1):165-175. doi:10.1007/s10029-020-02269-5*
[4] *Perez AJ, Campbell S. Inguinal Hernia Repair in Older Persons. J Am Med Dir Assoc. 2022;23(4):563-567. doi:10.1016/j.jamda.2022.02.008*
[9] *Li XY, Kong XM, Yang CH, et al. Global, regional, and national burden of ischemic stroke, 1990-2021: an analysis of data from the global burden of disease study 2021. EClinicalMedicine. 2024;75:102758. Published 2024 Jul 27. doi:10.1016/j.eclinm.2024.102758*
[18] *Liu X, Cheng R, Song Y, et al. Global burden of subarachnoid hemorrhage among adolescents and young adults aged 15-39 years: A trend analysis study from 1990 to 2021. PLoS One. 2024;19(12):e0316111. Published 2024 Dec 20. doi:10.1371/journal.pone.0316111*
[23] *Meier J, Stevens A, Berger M, et al. Comparison of Postoperative Outcomes of Laparoscopic vs Open Inguinal Hernia Repair. JAMA Surg. 2023;158(2):172-180. doi:10.1001/jamasurg.2022.6616*

*[25] Bali V, Adriano A, Byrne A, Akers KG, Frederickson A, Schelfhout J. Chronic cough: more than just a persistent cough: a systematic literature review to understand the impact of chronic cough on quality of life. Qual Life Res. 2024;33(4):903-916. doi:10.1007/s11136-023-03556-1*

*[26] Miyajima A. Inseparable interaction of the prostate and inguinal hernia. Int J Urol. 2018;25(7):644-648. doi:10.1111/iju.13717*

*[28] Kushner BS, Hamilton J, Han BJ, Sehnert M, Holden T, Holden SE. Geriatric assessment and medical preoperative screening (GrAMPS) program for older hernia patients. Hernia. 2022;26(3):787-794. doi:10.1007/s10029-021-02389-6*

*[35] Jansen CJ, Yielder PC. Evaluation of hernia of the male inguinal canal: sonographic method. J Med Radiat Sci. 2018;65(2):163-168. doi:10.1002/jmrs.275*

*[36] Howard R, Ehlers A, Delaney L, et al. Sex disparities in the treatment and outcomes of ventral and incisional hernia repair. Surg Endosc. 2023;37(4):3061-3068. doi:10.1007/s00464-022-09475-5*

*[38] Köckerling F, Koch A, Lorenz R. Groin Hernias in Women-A Review of the Literature. Front Surg. 2019;6:4. Published 2019 Feb 11. doi:10.3389/fsurg.2019.00004*

*[40] Whalen HR, Kidd GA, O'Dwyer PJ. Femoral hernias. BMJ. 2011;343:d7668. Published 2011 Dec 8. doi:10.1136/bmj.d7668*

*[46] Abozid H, Patel J, Burney P, et al. Prevalence of chronic cough, its risk factors and population attributable risk in the Burden of Obstructive Lung Disease (BOLD) study: a multinational cross-sectional study. EClinicalMedicine. 2024;68:102423. Published 2024 Jan 21. doi:10.1016/j.eclinm.2024.102423*

3. please define more about statistics

**Answer:** We sincerely appreciate your valuable feedback on the necessity of better defining the statistical methods. We have revised the Methods section to provide a more detailed explanation of the statistical approaches used in the GBD 2021 study, including age-standardized rates, the joinpoint regression model, the Socio-demographic Index, health inequality analysis, Bayesian age-period-cohort forecasting, and the statistical software employed.

*Line 109-180*

*“To ensure comparability across regions and populations, age-standardized indicators- including the ASIR, ASPR, and ASDR- were calculated using the World Health Organization standard population. This age standardization process adjusts for differences in population age structures, enabling meaningful temporal and geographical comparisons. The calculation methodology involves applying age-specific rates to the WHO standard population distribution, with demographic data derived from the GBD 2021 dataset. Participants were categorized into eight age groups (60–64, 65–69, 70–74, 75–79, 80–84, 85–89, 90–94, and 95–99 years) for analyzing the distribution of inguinal, femoral, and ventral hernias. Age-specific proportions were calculated by determining the ratio of cases within each age stratum relative to the corresponding standardized population cohort.[16] This stratification allows systematic examination of hernia epidemiology across distinct geriatric age segments while maintaining demographic comparability through standardized weighting procedures.*

*The* *joinpoint regression model was used to analyze the ASIR, ASPR, and ASDR temporal trends for the burden of inguinal, femoral, and abdominal hernia among older adults from 1990 to 2021. The method identifies points at which significant changes in trends occur and calculates the annual percentage change (APC) for each segment. The trend was quantified using the APC and the average annual percentage change (AAPC). APC represents the year-to-year change, while AAPC provides the average trend over a specified period [17].*

*The SDI is an aggregate measure formulated by GBD researchers to evaluate the socio-economic conditions impacting health indicators across different regions [14]. It is calculated as the geometric mean of indices for the total fertility rate under 25 (TFU25), mean education level for those aged 15 and above (EDU15+), and lag-distributed income (LDI) per capita [11]. The SDI values in the GBD 2021 study were scaled from 0 to 1. This composite indicator reflects a region's socio-economic health and progress, where higher SDI values indicate better socio-economic conditions and improved health outcomes. Regions are divided into 5 quintiles according to the 2021 SDI: low (0-0.466), low-middle (0.466-0.619), middle (0.619-0.712), high-middle (0.712-0 810), and high (0.810-1) [15]. These quintiles were utilized to explore disparities in disease burden across varying socio-demographic development levels. The ASIR, ASPR, and ASDR were employed in correlation analyses to investigate the relationship between the SDI and the burden of hernias among the elderly population. The analysis encompassed 204 countries and territories, as well as 21 regions. The strength and direction of the relationship between SDI and the burden of hernias among older adults were assessed by calculating Pearson correlation coefficients. A positive coefficient value indicates a direct relationship, whereas a negative value signifies an inverse relationship. This methodological approach enables a comprehensive evaluation of the association between socio-demographic development levels and hernia-related health outcomes in aging populations.*

*The ASRs and numbers of prevalence, incidence, and DALYs of inguinal, femoral, and abdominal hernia among older adults were extracted for inequality analysis. According to the World Health Organization Health Equity Assessment guidelines, the slope index of inequality (SII) and concentration index are two standard metrics for absolute and relative income-related inequality, which were used to assess the health inequalities of inguinal, femoral, and abdominal hernia among older adults across countries. The SII reflects the absolute difference in the above indicators between the lowest-SDI and highest-SDI countries. The higher absolute SII values indicate greater inequality. The concentration index was calculated using the Lorenz curve based on GDP per capita and the corresponding metrics of national burden, representing the area between the 45° line and the Lorenz curve. A negative index indicates that the burden is higher in low-income countries. Conversely, a positive index indicates a higher burden in high-income countries.*

*We employed the Bayesian Age-Period-Cohort (BAPC) model to analyze trends in hernia among the elderly population. This model decomposes temporal variations into age, period, and cohort effects while addressing the identifiability issues inherent in traditional APC models. Utilizing a Bayesian hierarchical approach, the BAPC model incorporates random walk priors to facilitate smooth transitions and employs hyperpriors to control variability, ensuring robust estimation even with sparse data. The posterior distributions generated by the BAPC model inherently account for variability and uncertainty associated with anomalous periods, ensuring that projections are not disproportionately influenced by short-term fluctuations. To enhance computational efficiency, we implemented the Integrated Nested Laplace Approximation (INLA), which provides accurate and scalable posterior inference. This framework enables the disentanglement of temporal effects, prediction of future trends, and quantification of uncertainty, making it a reliable methodology for large-scale analyses such as those conducted in the GBD studies.*

*ASRs were reported per 100,000 population, with data presented as values with 95% UI. Temporal trends were assessed using joinpoint software (version 5.0.2) from the National Cancer Institute. All statistical analyses and mapping were performed using R statistical software (version 4.3.3). A two-sided P value < 0.05 was set as the significance threshold.”*

**Reviewer #2:**

The study is based on data extracted from the Global Burden of Disease Study 2021 and the authors have studied the temporal trends in inguinal, femoral and abdominal hernia from 1990 to2021 and have also tried to show the future trends till 2035. The data mainly focusses on Age Standardized Incidence and Prevalence rates and age adjusted Disability life years and the impact of the SDI on these indices.

**Answer:** Thank you for your thoughtful review and for recognizing our efforts in analyzing the temporal trends of inguinal, femoral, and abdominal hernias using GBD 2021 data. We confirm that our study primarily focuses on age-standardized incidence, prevalence rates, and age-standardized disability-adjusted life years to ensure comparability across populations and over time. Additionally, we have assessed the impact of the SDI on these trends to highlight disparities in hernia burden globally.

**Reviewer #3:**

The manuscript is well written and technically sound. The statistical analysis used is sound and the database that was analysed is sound and globally accepted. In my opinion, this secondary sub-analysis of the GBD-2021 data to arrive at statistically significant and sound results will not only guide national and cross-border policy makers in resource allocation but also stimulates further regional research into the subject.

**Answer:** Thank you for your positive feedback and for recognizing the rigor of our analysis. We appreciate your acknowledgment of the statistical soundness and global relevance of our study. Our goal is to provide valuable insights for policymakers to aid in resource allocation and to encourage further regional research on hernia burden.

We are grateful for your support and look forward to contributing further to this important field.

**Reviewer #4:**

1. In this manuscript, the authors investigate the global trends and burden presented by inguinal, femoral, and abdominal hernias by analyzing data from the Global Burden of Disease Study 2021. Outcome measures include age-standardized incidence rates, prevalence and disability-adjusted life years (DALYs). Additionally, predictive analysis was performed on hernia incidence and prevalence up to the year 2035.
The Results section of the manuscript was difficult to follow, in part due to the excessive use of complicated abbreviations, and also in part due to frequent interruptions with Tables and Figure legends. The Discussion section, however, was beautifully written, and provided potential explanation to the unexpected data trends presented in the previous section.

**Answer:** Thank you for your thorough review, as well as your support and valuable suggestions. We sincerely apologize for any inconvenience caused by the interruption in Tables and Figure legends. Due to formatting constraints and limitations in the PDF preview, the readability of some elements may have been affected. Given the extensive use of abbreviations in this manuscript, we have included an Abbreviations section to provide a comprehensive reference for all abbreviations used, ensuring clarity for readers. To improve the continuity of the Results section, we have relocated Table 1 to the end of the manuscript. Furthermore, we have carefully revised the formatting and layout of figure legends to enhance readability.

*Line 548-563*

***Abbreviations*** *ASIR: Age-Standardized Incidence Rate
ASPR: Age-Standardized Prevalence Rate
ASDR: Age-Standardized Disability-Adjusted Life Year Rate
DALYs: Disability-Adjusted Life Years
AAPC: Average Annual Percentage Change
SDI: Socio-Demographic Index
UI: Uncertainty Interval
ICD: International Classification of Diseases
APC: Annual Percentage Change
BAPC: Bayesian Age-Period-Cohort
INLA: Integrated Nested Laplace Approximation
SII: Slope Index of Inequality
TFU25: Total Fertility Rate Under 25
EDU15+: Mean Education Level for Those Aged 15 and Above
LDI: Lag-Distributed Income*

2. The manuscript places a heavy emphasis on the burden of hernia on older adults. How was "older adults" defined in this study?

**Answer:** We sincerely appreciate your insightful comments. In our study, we define older adults as individuals aged 60 years and above, aligning with widely accepted definitions in public health and GBD studies. For instance, the WHO Report on Aging and Health and several GBD-based studies adopt 60 years as the threshold for aging. Additionally, a study in Nature Aging (Shen et al.) suggests that physiological decline accelerates from this age. Defining older adults as 60 years and above ensures consistency with global datasets and facilitates meaningful comparisons with aging-related health research. In the methods section, we have provided a more detailed definition of the older adults.

*Line 98-103*

*“Based on the World Health Organization's "World Report on Ageing and Health" and prior research on the disease burden among older adults using the GBD framework, our study defines "older adults" as individuals aged 60 years and older [13,16]. This definition is consistent with global public health standards and widely recognized in studies examining ageing and its associated health impacts.”*

3. What remains puzzling is that the data indicates a consistent downward trend in age-specific incidence and prevalence, up until the onset of the covid pandemic (2019-2020) across all sociodemographic categories. And yet, there was a dramatic increase in the total number of cases since the beginning of the study period in 1990. Please provide an explanation for this discrepancy.

**Answer:** We are grateful for your thoughtful observations. As you pointed out, while the ASIR for inguinal hernia among the elderly population has been declining from 1990 to 2019, the absolute number of cases has been steadily rising. This intriguing phenomenon can be largely attributed to the global population aging. Over this 30-year period, the population aged 60 and above nearly doubled, from 538 million to 1.05 billion. Moreover, due to the deterioration of physical function and increased susceptibility to chronic conditions in the elderly, this demographic is more prone to developing hernias, leading to the continued rise in the total number of cases despite the decline in age-specific incidence and prevalence. We have expanded our discussion section to provide a more thorough explanation for this intriguing phenomenon.

*Line 403-425*

*“Our study found that, although the ASIR has been steadily declining over time, particularly between 1990 and 2019, the absolute number of hernia cases among older adults has continued to rise. This disparity can be largely attributed to the accelerating global population aging. Over the 30-year period from 1990 to 2019, the global population increased from 5.32 billion to 7.70 billion [12]. According to data published by the World Health Organization, the population of individuals aged 60 years and above nearly doubled, rising from 538 million to 1.05 billion. As a result, despite advancements in medical care leading to a reduction in ASIR, the significant expansion of the elderly population base has driven an overall increase in hernia cases among older adults. Previous studies have identified aging as a key risk factor for hernia development [27–28]. With advancing age, abdominal wall muscles progressively weaken and lose elasticity, while the structure and function of connective tissues deteriorate [29]. Additionally, elderly individuals are more susceptible to chronic conditions such as persistent coughing, constipation, and benign prostatic hyperplasia, which can contribute to increased intra-abdominal pressure, further elevating their susceptibility to hernia formation. Some studies suggest that individuals experience a precipitous decline in physiological function around the age of 60 [30], which may explain the rising number of hernia cases in this demographic. Given current demographic projections, the global elderly population is expected to increase by approximately 20% by 2050. If this trend continues, the burden of hernia among older adults will likely escalate further. Without proactive health policy interventions, the rising number of hernia cases in the aging population is expected to pose substantial challenges to healthcare systems worldwide.”*

4. The most questionable portion of this manuscript is in its predictive modeling. Again, consistent declines in incidence of prevalence of hernias was shown, up until the onset of the covid pandemic which began in 2019-2020. Since then, there has been a steady increase in incidence/prevalence.
The predictive modeling seems to show that a continued upward trend is expected until year 2035. Please explain how the data collected during the covid years were handled in predictive modeling, and whether the trends observed over the past few years are expected to continue.

**Answer:** Thank you for your valuable feedback and insightful comments. Regarding the forecasting methodology, we utilized the BAPC (Bayesian Age-Period-Cohort) model, which disentangles the cyclical effects from the age and cohort effects. This allowed us to isolate the potential impacts of the pandemic period. The BAPC model's posterior distributions inherently capture the variability and uncertainty associated with these anomalous COVID-19 time, thereby preventing short-term fluctuations from disproportionately influencing the predictions. The BAPC model emphasizes long-term historical trends while seamlessly integrating the most recent data. This approach minimizes the influence of short-term disruptions, while still reflecting the current incidence and prevalence trends. The patterns observed during the pandemic period are not expected to significantly alter the long-term trajectory, and we have provided a detailed description of the BAPC model and our handling of the COVID-19 data in the methods section.

You're correct that as noted, the BAPC model indicates that as the forecasting horizon extends, the confidence intervals around future disease burden projections may widen, potentially reducing the reliability of the predictions. This limitation of the forecasting analysis has been detailed in the discussion section.

Based on the current forecasting, the ASIR and ASPR have been increasing since the pandemic years. As the population continues to age further, with a sustained rise in the incidence of chronic diseases among the elderly, this trend of ASIR and ASPR is likely to persist in the future. However, whether this trend will continue to hold in the future remains uncertain. We will need to perform ongoing recalculations as the GBD data is updated, as the future developments in healthcare and the long-term impacts of COVID-19 on healthcare and the elderly population's health are still unknown. Thank you again for your insightful comments and the opportunity to provide this response. Your feedback will be invaluable in enhancing the quality and robustness of our analysis.

*Line 162-175*

*“We employed the Bayesian Age-Period-Cohort (BAPC) model to analyze trends in hernia among the elderly population [7]. This model decomposes temporal variations into age, period, and cohort effects while addressing the identifiability issues inherent in traditional APC models. Utilizing a Bayesian hierarchical approach, the BAPC model incorporates random walk priors to facilitate smooth transitions and employs hyperpriors to control variability, ensuring robust estimation even with sparse data. The posterior distributions generated by the BAPC model inherently account for variability and uncertainty associated with anomalous periods, ensuring that projections are not disproportionately influenced by short-term fluctuations. To enhance computational efficiency, we implemented the Integrated Nested Laplace Approximation (INLA), which provides accurate and scalable posterior inference. This framework enables the disentanglement of temporal effects, prediction of future trends, and quantification of uncertainty, making it a reliable methodology for large-scale analyses such as those conducted in the GBD studies [18].”*

*Line 497-499*

*“Compared to GBD 2019, the GBD 2021 dataset provides information on the post-COVID-19 period. To enable more accurate forecasting of future inguinal hernia burden among the elderly, we employed the BAPC model in our analysis.”*

*Line504-506*

*“As the population continues to age further, with a sustained rise in the incidence of chronic diseases among older adults [46], this trend of ASIR and ASPR is likely to persist in the future.”*

*Line 517-519*

*“The GBD2021 includes data on elderly hernia cases related to COVID-19, and the impacts of COVID-19 on the healthcare system and elderly patients may both affect the accuracy of predictive analysis.”*
