# Supplementary material for: Global burden and future trends of inguinal, femoral, and abdominal hernia in older adults: A systematic analysis from the Global Burden of Disease Study 2021
Source: PLoS One. 2025 Jun 6;20(6):e0323790. doi: 10.1371/journal.pone.0323790 (PMC12143580; doi:10.1371/journal.pone.0323790)
Supplement: Attachment — Submitted filename: PLOS oneREVIEWER response.docx [file pone.0323790.s003.docx]

**REVIEWER’S RESPONSE**

This paper discussed the **global, regional, and national burden** of **inguinal, femoral, and abdominal hernias** among **older adults** (aged 60+) from **1990 to 2021**. The paper used data from the **Global Burden of Disease (GBD) 2021**, by analyzing the trends in **incidence, prevalence, and Disability-Adjusted Life Years (DALYs)**, while exploring **health inequalities**based on **Socio-Demographic Index (SDI)** levels.

### **Despite that the manuscript is riched in information and provioded extensive details about different type of hernia, however, below are some of observation and concerns about the manuscript.**

**METHODS**

#### 1. The methods section is dense and difficult to navigate, with long paragraphs and repetitive content. Like example of ASR on line 149-161 almost saying the same thing with line 176-180

#### 2. The use of INLA in the BAPC model on line 499 is mentioned without sufficient justification of the use in the study.

#### 3. There is no mention of potential data biases or limitations, such as underreporting in certain regions (especially in Afrtica and Southeast Asian)or the accuracy of ICD codes across all countries that was mentionerd.

#### 4. While SII (Line 149-161) and concentration indices are applied, the rationale for choosing these metrics over others is missing. Additionally, the description of the Lorenz curve (Line 157-159) could be clearerfor re use by other authors.

#### **5.** The description of joinpoint regression (Line 122, 199) and BAPC lacks mention of cross-validation or sensitivity analyses.

#### 6. There’s no mention of ethical approval or data usage agreements, even though GBD data (line 512) is publicly available.

**RESULT**

### 1. The results section is densely packed with figures, percentages, and statistical outcomes, leading to information overload. It makes it hard to extract key insights quickly.

### 2. There is limited discussion on why certain regions (like europe, some part of Asia and Africa) have higher or lower burdens or why trends have changed (e.g., why East Asia saw an ASIR increase).

### 3. Abbreviations like ASIR, ASPR, and ASDR are defined multiple times, while others (e.g., APCC) are introduced late or without clarity.

**DISCUSSION**

### 1. Several sections repeat information, especially statistics on ASIR, ASPR, and ASDR trends. For example, the decline in these rates is mentioned multiple times (e.g., lines 366-368 and 383-385).

### 2. Although, the paper notes disparities between SDI regions, the analysis lacks depth. For instance, the reasons behind persistent high burdens in low-SDI areas are mentioned briefly without exploring systemic issues like healthcare access or cultural factors.

### 3. The paper presents many AAPC and APC values but often lacks a deeper interpretation of their implications.

### 4. Many sentences are long and dense, affecting readability (e.g., lines 405-411).

**REFERENCES**

1. Some references use “*et al.*” after several authors ([3], [9]), while others list all authors regardless of length ([6], [26]).
2. Some journal titles are abbreviated (e.g., Int J Surg) while others are written fully (e.g., Journal of the American Medical Directors Association in [4]).
3. Some include full dates (e.g., [9], [18]), while others use only the year and month (e.g., [1], [7]).
4. Inconsistent usage of “doi:” vs. “DOI” and irregular spacing. kindly check this please.

Overall, the paper was written in a robust manner and I believe it will add knowledge to the field of hernia in medical sciences.
