# Supplementary material for: Global burden and future trends of inguinal, femoral, and abdominal hernia in older adults: A systematic analysis from the Global Burden of Disease Study 2021
Source: PLoS One. 2025 Jun 6;20(6):e0323790. doi: 10.1371/journal.pone.0323790 (PMC12143580; doi:10.1371/journal.pone.0323790)
Supplement: Attachment — Submitted filename: Response_to_Reviewers_auresp_2.docx [file pone.0323790.s005.docx]

**5 April 2025**

Lovenish Bains, MS, FNB, FACS, FRCS (Glas), FICS, FIAGES, PhD

Academic Editor

PLOS ONE

Dear Editor and Reviewers:

We really appreciate the critical reading of our manuscript entitled “Global burden and future trends of inguinal, femoral, and abdominal hernia in older adults: a systematic analysis from the Global Burden of Disease Study 2021” (Manuscript Number: PONE-D-24-46904R2). We thank you for all the valuable suggestions received from the reviewers and editor. We have carefully considered the comments and have revised the manuscript accordingly. The responses to the comments are listed one by one as follows. (Please see below, the responses to the editor and reviewers are represented in red font, while the modifications made to the original text are shown in blue font.) We are looking forward to hearing about your decision.

**REVIEWER’S RESPONSE**

This paper discussed the **global, regional, and national burden** of **inguinal, femoral, and abdominal hernias** among **older adults** (aged 60+) from **1990 to 2021**. The paper used data from the **Global Burden of Disease (GBD) 2021**, by analyzing the trends in **incidence, prevalence, and Disability-Adjusted Life Years (DALYs)**, while exploring **health inequalities** based on **Socio-Demographic Index (SDI)** levels.

### **Despite that the manuscript is riched in information and provioded extensive details about different type of hernia, however, below are some of observation and concerns about the manuscript.**

**METHODS**

#### 1. The methods section is dense and difficult to navigate, with long paragraphs and repetitive content. Like example of ASR on line 149-161 almost saying the same thing with line 176-180.

**Answer：**Thank you for your valuable comment. As you rightly pointed out, the Methods section was relatively long and contained some redundant content. In response, we have removed repetitive descriptions to improve clarity and conciseness. Additionally, we have added subheadings to facilitate navigation and enhance the structural organization of the section.

#### 2. The use of INLA in the BAPC model on line 499 is mentioned without sufficient justification of the use in the study.

**Answer：**Thank you for your constructive comment. As you rightly pointed out, although we applied the INLA framework in the BAPC model, we did not provide sufficient details on its implementation to justify its use in our study. In response, we have revised the Methods section to explicitly describe how INLA was applied in the construction of the BAPC model. Specifically, the BAPC model in our analysis was implemented using the INLA approach to approximate the marginal posterior distributions. Compared to traditional Bayesian methods based on Markov Chain Monte Carlo (MCMC), INLA avoids common issues related to convergence and mixing, and allows for more efficient and accurate prediction.

*Line 187-202*

*“We employed a Bayesian age-period-cohort (BAPC) model to analyze the temporal trends of hernia among older adults[22]. This model is based on the age-period-cohort model, which makes predictions based on the assumption that the effects of age, period, cohort are approximated at adjacent time points in the same study population and within the same study period. To smooth the prior estimates of age, period, and cohort effects, we specified second-order random walk (RW2) priors, which allow for flexible yet structured temporal evolution[18]. The BAPC model was implemented using the Integrated Nested Laplace Approximation (INLA) method to approximate the marginal posterior distributions. INLA enables efficient and accurate Bayesian inference for latent Gaussian models, and effectively circumvents the convergence and mixing issues commonly encountered in traditional Bayesian approaches based on Markov Chain Monte Carlo (MCMC) techniques. Compared to MCMC, INLA typically provides reliable posterior estimates with substantially reduced computational burden[23]. By incorporating both historical data and prior distributions, the BAPC model with INLA allows for more accurate estimation and projection of hernia incidence in older populations while explicitly accounting for age, period, and cohort effects.”*

#### 3. There is no mention of potential data biases or limitations, such as underreporting in certain regions (especially in Afrtica and Southeast Asian) or the accuracy of ICD codes across all countries that was mentionerd.

**Answer：**Thank you for your valuable comment. As you correctly pointed out, underreporting of cases and inaccurate coding of disease diagnoses may occur in some low-resource countries in Africa and Southeast Asia, where health information systems are underdeveloped. These factors may introduce data bias and affect the accuracy of disease burden estimates in these regions. In response, we have added a corresponding statement to the limitations section of the Discussion to acknowledge this potential source of uncertainty in the GBD data.

*Line 547-554*

*“In particular, low- and middle-income regions such as sub-Saharan Africa and parts of Southeast Asia may face challenges due to underdeveloped health information systems, resulting in underreporting of cases and deaths, incomplete vital registration, and inconsistent use of ICD coding by healthcare providers and institutions. Although the GBD study applies garbage code redistribution, covariate-based modeling, and rigorous data quality assessments to address these issues, residual uncertainties may still affect the accuracy of disease burden estimates in certain regions.”*

#### 4. While SII (Line 149-161) and concentration indices are applied, the rationale for choosing these metrics over others is missing. Additionally, the description of the Lorenz curve (Line 157-159) could be clearer for re-use by other authors.

**Answer：**Thank you for your insightful comments. In response, we have revised the Methods section to clarify the rationale for using both the Slope Index of Inequality (SII) and the Concentration Index (CI) in our analysis. These two complementary metrics provide a comprehensive assessment of health inequality across socioeconomic gradients. Additionally, since the CI is derived from the Lorenz concentration curve, we have added a detailed description of the Lorenz concentration curve to enhance clarity for readers unfamiliar with this method.

*Line 160-185*

*“To quantify and characterize the socioeconomic inequality in the burden of elderly hernias across countries, we employed two complementary indices: the Slope Index of Inequality (SII) and the Concentration Index (CI). These indices were selected due to their methodological robustness and their ability to capture both the magnitude and direction of inequality across the full socioeconomic spectrum.*

*The SII is an absolute measure of inequality derived from a weighted linear regression model, where countries are ranked by their SDI and weighted by population size. It estimates the absolute difference in the health burden between the hypothetical lowest and highest ends of the socioeconomic hierarchy. By incorporating data from all countries rather than only comparing extreme groups, the SII provides a comprehensive assessment of the socioeconomic gradient in health outcomes. The higher absolute SII values indicate greater inequality[19]. The CI was calculated by fitting a Lorenz concentration curve to the observed cumulative relative distribution of the populations ranked by SDI and the prevalence of disease, as well as numerically integrating the area under the curve. A negative index indicates that the burden is higher in low-income countries. Conversely, a positive index indicates a higher burden in high-income countries[20]. The Lorenz concentration curve is used to assess socioeconomic-related inequality in health outcomes. The X-axis represents the cumulative population ranked by a socioeconomic indicator, while the Y-axis shows the cumulative proportion of the health variable. The 45-degree line indicates perfect equality. A curve below the line suggests the burden is concentrated among lower-SDI populations; above indicates concentration among higher-SDI groups. The degree of deviation from the equality line reflects the magnitude of inequality. This curve is often used alongside the concentration index to quantify and interpret health disparities across socioeconomic gradients[21].”*

#### 5. The description of joinpoint regression (Line 122, 199) and BAPC lacks mention of cross-validation or sensitivity analyses.

**Answer：**Thank you for your insightful comment. We fully agree that model validation through techniques such as cross-validation or sensitivity analysis is essential to enhance the credibility and robustness of statistical models, especially in predictive epidemiology. In response, we have revised the Methods section to clarify the model selection procedures and added sensitivity analysis to test the robustness of our findings.

For Joinpoint regression, we used the Joinpoint software (version 5.0.2), which employs a Monte Carlo permutation method to identify the optimal number of joinpoints without overfitting. While cross-validation is not a standard component of the NCI Joinpoint framework, the permutation test and model fit statistics help ensure model validity. Additionally, we conducted sensitivity analyses by varying the maximum number of allowed joinpoints (from 2 to 4) and confirmed that the overall temporal trends and AAPC estimates remained consistent.

For the BAPC model, we implemented it using the BAPC package in R, which is based on INLA. We performed sensitivity analyses by altering prior distributions for age, period, and cohort effects. The projected trends remained stable across different prior specifications, suggesting the robustness of our projections. These details are now included in the revised Methods.

*Line 160-185*

*“Joinpoint regression analysis partitions the continuous time-series data into multiple linear segments, each representing a statistically distinct trend over time. Within each segment, a separate linear model is fitted to estimate the temporal trend, and the number and location of joinpoints are determined using a Monte Carlo permutation test. This test assesses whether adding a joinpoint significantly improves model fit[15]. For each segment, the model calculates the annual percent change (APC) and its 95% confidence interval to quantify the direction and magnitude of the trend. The average annual percent change (AAPC) is then computed to summarize the overall trend across the entire study period[16].”*

*Line 191-202*

*“To smooth the prior estimates of age, period, and cohort effects, we specified second-order random walk (RW2) priors, which allow for flexible yet structured temporal evolution[18]. The BAPC model was implemented using the Integrated Nested Laplace Approximation (INLA) method to approximate the marginal posterior distributions. INLA enables efficient and accurate Bayesian inference for latent Gaussian models, and effectively circumvents the convergence and mixing issues commonly encountered in traditional Bayesian approaches based on Markov Chain Monte Carlo (MCMC) techniques. Compared to MCMC, INLA typically provides reliable posterior estimates with substantially reduced computational burden[23]. By incorporating both historical data and prior distributions, the BAPC model with INLA allows for more accurate estimation and projection of hernia incidence in older populations while explicitly accounting for age, period, and cohort effects.”*

#### 6. There’s no mention of ethical approval or data usage agreements, even though GBD data (line 512) is publicly available.

**Answer：**Thank you for your thoughtful comment. In response, we have added a statement regarding ethical approval and data use agreements in the Methods section to clarify compliance with relevant ethical standards and data access policies.

*Line 208-216*

*“Ethical Statement*

*This study is based on publicly available data from the GBD 2021 study, which is coordinated by the Institute for Health Metrics and Evaluation (IHME). All data used in this analysis are aggregated, de-identified, and available in the public domain (https://ghdx.healthdata.org/gbd-2021), this study meets the criteria for exemption from full ethical review according to the guidelines provided by Shanxi Provincial People’s Hospital Ethics Committee and the University of Washington’s IRB, which has oversight over the GBD project. The study was conducted in accordance with the Declaration of Helsinki and relevant guidelines and regulations.”*

**RESULT**

### 1. The results section is densely packed with figures, percentages, and statistical outcomes, leading to information overload. It makes it hard to extract key insights quickly.

**Answer：**Thank you for your professional insight. To facilitate easier extraction of key information for readers, we have removed some numerical values and detailed statistical results from the Results section and instead presented them in the tables. This modification improves the readability and conciseness of the main text while ensuring that all essential data remain accessible.

*Line 227-232*

*“Between 1990 and 2021, the global burden of hernias among older adults showed an overall decline in the ASIR, ASPR, and ADSR. The ASIR decreased from 190.28 to 134.87 per 100,000 population (AAPC = -1.12, 95% CI: -1.24 to -0.99, P < 0.001), the ASPR declined from 516.42 to 330.86 per 100,000 (AAPC = -1.44, 95% CI: -1.48 to -1.40, P < 0.001), and the ASDR dropped from 127.62 to 76.23 per 100,000 (AAPC = -1.64, 95% CI: -1.77 to -1.51, P < 0.001).”*

### 2. There is limited discussion on why certain regions (like europe, some part of Asia and Africa) have higher or lower burdens or why trends have changed (e.g., why East Asia saw an ASIR increase).

**Answer：**Thank you for your valuable comment. To improve the completeness of our manuscript, we have added a discussion of the potential factors contributing to the increase or decrease in the burden of inguinal hernia among older adults across different regions. This addition aims to provide a more comprehensive interpretation of the observed regional trends and to enhance the contextual understanding of our findings.

*Line 504-513*

*“At region level, the consistently high ASIR in Central and Western Europe may reflect better case detection and diagnostic capacity, especially among older adults undergoing routine health evaluations. In contrast, the persistently high ASPR and ASDR in Central Latin America likely indicate a combination of limited access to timely surgical care and longer disease duration due to treatment delays. The recent increase in ASIR in East Asia is particularly noteworthy. This trend may be partly explained by rapid population aging, increased health service utilization, and improved case detection in countries such as China, where health system infrastructure has expanded significantly in recent decades[50]. However, this increase could also reflect shifts in health-seeking behavior or greater awareness of hernias among older individuals.”*

### 3. Abbreviations like ASIR, ASPR, and ASDR are defined multiple times, while others (e.g., APCC) are introduced late or without clarity.

**Answer：**Thank you for your careful observation. We have removed the redundant definitions of ASIR, ASPR, and ASDR to avoid repetition. In addition, "APCC" was a typographical error; the correct term is "AAPC" (average annual percent change), and this has been corrected throughout the manuscript.

**DISCUSSION**

### 1. Several sections repeat information, especially statistics on ASIR, ASPR, and ASDR trends. For example, the decline in these rates is mentioned multiple times (e.g., lines 366-368 and 383-385).

**Answer：**Thank you for your helpful comment. In response, we have removed redundant information from the Discussion section and omitted certain statistical details to improve clarity and conciseness. These revisions aim to enhance the overall readability and focus of the discussion.

### 2. Although, the paper notes disparities between SDI regions, the analysis lacks depth. For instance, the reasons behind persistent high burdens in low-SDI areas are mentioned briefly without exploring systemic issues like healthcare access or cultural factors.

**Answer：**Thank you for your professional insight. In response, we have expanded the Discussion section to include potential reasons underlying the persistently high burden of disease in low-SDI regions. This addition aims to deepen the interpretation of our findings and provide a more comprehensive understanding of regional disparities.

*Line 487-495*

*“This reflects deeply rooted structural disparities within health systems. Although the global burden of hernias has shown a declining trend, low-SDI regions continue to face substantial challenges in the timely diagnosis and surgical management of hernias among older adults. A key contributing factor is the limited access to safe, affordable, and timely surgical care. According to the Lancet Commission on Global Surgery, more than 5 billion people worldwide lack access to essential surgical services, with the majority residing in low-SDI settings[47]. In these regions, surgical infrastructure remains underdeveloped, and there is a critical shortage of trained surgeons, anesthesiologists, and perioperative personnel.”*

### 3. The paper presents many AAPC and APC values but often lacks a deeper interpretation of their implications.

**Answer：**Thank you for your valuable comment. In response, we have added a more detailed explanation of the AAPC and APC values in the Discussion section to enhance readers’ understanding of the temporal trends presented in our study.

*Line 411-414*

*“Our study showed that the ASIR, ASPR and ASDR for hernias in older adults decreased* *in 2021 compared to 1990, with AAPC values of -1.12, -1.44, and -1.64, respectively. These negative AAPC values indicate sustained, long-term reductions in both the burden and progression of hernias globally.”*

*Line 448-452*

*“Surgical delays and reduced medical services during COVID-19 lockdowns ultimately led to the progression of hernias and an increase in ASIR (APC= 1.87, 95% CI: 0.96 to 2.79, P < 0.001) and ASPR (APC= 0.49, 95% CI: 0.18 to 0.80, P = 0.004)[37,38]. The pandemic also disrupted the management of chronic diseases, exacerbating risk factors associated with hernias and promoting their progression[39].”*

### 4. Many sentences are long and dense, affecting readability (e.g., lines 405-411).

**Answer：**Thank you for your valuable suggestion. To improve the readability of the Discussion section, we have streamlined several lengthy paragraphs by condensing the content and improving the structure. These revisions aim to enhance clarity while preserving the key points of the discussion.

*Line 430-444*

*“Our study found that, although the ASIR has been steadily declining over time, particularly between 1990 and 2019, the absolute number of hernia cases among older adults has continued to rise. This apparent contradiction is primarily driven by global population aging. Between 1990 and 2019, the world population grew from 5.32 to 7.70 billion[12], while the number of people aged 60 years and older nearly doubled, from 538 million to 1.05 billion. Aging is a well-established risk factor for hernia development[32,33], as age-related weakening of abdominal muscles and connective tissue increases susceptibility[34]. Moreover, common comorbidities in older adults, such as chronic cough, constipation, and prostatic hypertrophy, can raise intra-abdominal pressure, further elevating hernia risk. Physiological decline tends to accelerate after age 60, making this population particularly vulnerable[35]. Given projections that the global elderly population will increase by 20% by 2050, the overall burden of hernias is expected to continue rising. Without timely health system responses, this trend may place increasing strain on surgical capacity and elderly care services worldwide.”*

**REFERENCES**

1. Some references use “*et al.*” after several authors ([3], [9]), while others list all authors regardless of length ([6], [26]).

**Answer：**Thank you for your helpful suggestion. We have revised the reference formatting accordingly, and now use “et al.” after listing a few authors, in accordance with the journal's guidelines and standard referencing style.

2. Some journal titles are abbreviated (e.g., Int J Surg) while others are written fully (e.g., Journal of the American Medical Directors Association in [4]).

**Answer：**Thank you for your valuable comment. We have revised the reference formatting and standardized it using the abbreviated style, in accordance with the journal’s requirements.

3. Some include full dates (e.g., [9], [18]), while others use only the year and month (e.g., [1], [7]).

**Answer：**Thank you for your helpful suggestion. We have revised the reference formatting and have standardized the date presentation to include only the year and month, in accordance with the journal’s style requirements.

4. Inconsistent usage of “doi:” vs. “DOI” and irregular spacing. kindly check this please.

**Answer：**Thank you for your constructive suggestion. We have revised the reference formatting in accordance with the journal’s requirements.

Overall, the paper was written in a robust manner and I believe it will add knowledge to the field of hernia in medical sciences.
